# Prevalence and predictors of sustained remission/low disease activity after discontinuation of induction or maintenance treatment with tumor necrosis factor inhibitors in rheumatoid arthritis: a systematic and scoping review

**DOI:** 10.1186/s13075-023-03199-0

**Published:** 2023-11-20

**Authors:** Michael M. Ward, Nima Madanchi, Ali Yazdanyar, Nehal R. Shah, Florina Constantinescu

**Affiliations:** 1 Intramural Research Program, National Institute of Arthritis and Musculoskeletal and Skin Diseases, National Institutes of Health, Building 10CRC, Room 4-1339, 10 Center Drive, Bethesda, MD 20892-1468 USA; 2 Division of Rheumatology, Allergy, and Immunology, Department of Internal Medicine, Virginia Commonwealth University, Richmond, VA USA; 3 Current address: Division of Rheumatology, Johns Hopkins University School of Medicine, Baltimore, MD USA; 4 Department of Emergency and Hospital Medicine, Lehigh Valley Hospital-Cedar Crest, Allentown, PA USA; 5 Morsani College of Medicine, University of South Florida, Tampa, FL USA; 6 Current address: Division of Hospital Medicine, University of Pennsylvania Perelman School of Medicine, Philadelphia, PA USA; 7 Division of Rheumatology, MedStar Washington Hospital Center, Washington, DC USA

**Keywords:** Rheumatoid arthritis, Tumor necrosis factor inhibitor, Remission

## Abstract

**Background:**

To determine the prevalence of sustained remission/low disease activity (LDA) in patients with rheumatoid arthritis (RA) after discontinuation of tumor necrosis factor inhibitors (TNFi), separately in induction treatment and maintenance treatment studies, and to identify predictors of successful discontinuation.

**Methods:**

We performed a systematic literature review of studies published from 2005 to May 2022 that reported outcomes after TNFi discontinuation among patients in remission/LDA. We computed prevalences of successful discontinuation by induction or maintenance treatment, remission criterion, and follow-up time. We performed a scoping review of predictors of successful discontinuation.

**Results:**

Twenty-two induction-withdrawal studies were identified. In pooled analyses, 58% (95% confidence interval (CI) 45, 70) had DAS28 < 3.2 (9 studies), 52% (95% CI 35, 69) had DAS28 < 2.6 (9 studies), and 40% (95% CI 18, 64) had SDAI ≤ 3.3 (4 studies) at 37–52 weeks after discontinuation. Among patients who continued TNFi, 62 to 85% maintained remission. Twenty-two studies of maintenance treatment discontinuation were also identified. At 37–52 weeks after TNFi discontinuation, 48% (95% CI 38, 59) had DAS28 < 3.2 (10 studies), and 47% (95% CI 33, 62) had DAS28 < 2.6 (6 studies). Heterogeneity among studies was high. Data on predictors in induction-withdrawal studies were limited. In both treatment scenarios, longer duration of RA was most consistently associated with less successful discontinuation.

**Conclusions:**

Approximately one-half of patients with RA remain in remission/LDA for up to 1 year after TNFi discontinuation, with slightly higher proportions in induction-withdrawal settings than with maintenance treatment discontinuation.

**Supplementary Information:**

The online version contains supplementary material available at 10.1186/s13075-023-03199-0.

## Background

With recent advances in therapy, the current goal of treatment of rheumatoid arthritis (RA) is clinical remission. While 30% of patients treated with conventional synthetic disease-modifying medications (csDMARDs) achieve remission, up to 50% of those treated with tumor necrosis factor inhibitors (TNFi) in a treat-to-target strategy achieve remission at 6 to 12 months, with better physical functioning, less radiographic damage, and lower risks of work loss [1–3].

With this growing population of patients, new questions have arisen about the most appropriate regimen to maintain remission. In particular, for patients treated with TNFi in combination with csDMARDs, what are the relative benefits and risks of continuing versus discontinuing TNFi? Discontinuation of TNFi could avoid potential overtreatment and eliminate associated costs and risks of toxicities [4]. Also, because patients in remission may experiment with unsupervised drug holidays, supervised discontinuation may improve overall adherence [5, 6]. However, TNFi discontinuation entails risks of increased RA activity. Previous reviews have reported that 40 to 50% of patients could maintain remission at least short-term after stopping TNFi, but loss of remission was 1.3 to 6.7 times more likely compared to those who continued treatment [4, 7–12].

TNFi discontinuation may take place in two clinical contexts: when remission has been achieved after short-term use of TNFi as induction therapy (i.e., an induction-withdrawal approach), or more commonly, among patients in stable remission after long-term treatment (i.e., maintenance discontinuation). Viewed in the Population-Intervention-Comparator-Outcome (PICO) framework, these populations differ. It is important to examine these populations separately because the duration of RA, recency of active RA, and duration of remission may influence the success of TNFi discontinuation [12]. Previous reviews have not distinguished these different clinical scenarios, even though information on each group is needed for accurate patient counseling.

That about one-half of patients can successfully discontinue TNFi suggests that there may be subsets of patients with either higher or lower likelihoods of success. If these subsets could be identified, TNFi discontinuation could be more effectively targeted. The most consistent predictors of successful TNFi discontinuation have been the depth of remission and early RA [13]. Associations with other clinical features, particularly biomarkers, are less certain [12–15]. Whether predictors differ between patients stopping induction treatment or maintenance treatment is unknown.

Our goals were as follows: (1) to perform a systematic review of the prevalence of remission after TNFi discontinuation, separately in patients receiving induction therapy or stopping maintenance treatment, and (2) to perform a scoping review of predictors of remission in these two populations. We focused on TNFi discontinuation because this is currently the most common treatment de-escalation decision in RA [16].

## Methods

We performed two related literature reviews: a systematic review of the prevalence of sustained remission/low disease activity (LDA) after discontinuation of TNFi treatment in patients with RA (and when available, comparison to continuation of TNFi), and a scoping review of predictors of continued remission/LDA after TNFi discontinuation [17]. We examined both questions following a written protocol, which was registered at the Center for Open Science (osf.oi/etzav). We followed the Preferred Reporting Items for Systematic Reviews and Meta-analyses 2020 recommendations (Supplement) [18].

### Literature searches

We searched five bibliographic databases for relevant studies in any language published from January 1, 2005, to May 1, 2022: PubMed/MEDLINE, Embase, Web of Science, Cochrane Central Register of Controlled Trials, and Cochrane Reviews. We did not search before 2005 because discontinuation strategies were not used earlier. Search terms included “rheumatoid arthritis,” “tumor necrosis factor inhibitors,” individual medication names, “remission” or “low disease activity,” and “discontinuation” or “withdrawal” (Supplemental Table 1). We used EndNote20 for citation management. For the scoping review, one author also searched abstracts of congresses of the American College of Rheumatology and European League Against Rheumatism from 2010 to 2022 and Google through May 2023.

### Study inclusion

Two authors independently reviewed the search results for relevant articles, first by title/abstract and subsequently full-text review. Discrepancies were resolved by discussion. We included full-length articles, reviews, conference abstracts, and trial registrations to identify primary articles and for the scoping review, but limited the systematic review to full-length articles. We included randomized controlled trials, single-arm trials, and observational studies that examined adults with RA who were in remission/LDA while on treatment with TNFi, and that reported patients’ remission status following discontinuation of TNFi treatment. We included articles regardless of the stringency of remission or RA activity index used, on the premise that investigators judged that RA activity was low enough that TNFi discontinuation was a reasonable consideration. Some studies had a controlled trial design to address a different primary question, but included TNFi discontinuation during follow-up as a secondary aim. We considered these as observational studies if TNFi discontinuation was not randomized.

We excluded cross-sectional studies, studies of other diseases or children or animals, case reports, letters, duplicate articles, and abstracts subsequently published as full-length articles. We also excluded studies of discontinuation of csDMARDs or other biologics unless the article included stratified data on TNFi. We excluded TNFi tapering studies and tapering arms of multi-arm trials (Supplemental Table 2). We focused on discontinuation rather than tapering, as tapering regimens vary, and discontinuation provides greater contrast to identify predictors. When more than one article was based on the same cohort, we included the article most relevant to the systematic or scoping review.

For the scoping review, we included full-length articles or conference abstracts that examined predictors of sustained remission/LDA after TNFi discontinuation. Predictors could be either clinical, imaging, or biological markers. We allowed studies that included patients who discontinued other biologics, provided that most patients used TNFi, and allowed studies that reported predictors of remission in the entire cohort (i.e., not limited to those who discontinued TNFi).

### Data extraction

For the systematic review, two authors independently extracted data on RA activity at the time of TNFi discontinuation, remission/LDA criteria, prevalence of remission/LDA during follow-up, and outcomes of re-treatment, using a standardized format. Two authors also independently assessed study quality, using the Cochrane Risk of Bias 2 (ROB2) tool for controlled trials and the Risk Of Bias In Non-randomized Studies of Interventions (ROBINS-I) tool for other studies [19, 20]. Results were compared and discrepancies resolved by discussion. For the scoping review, data on predictors and measures of association were extracted by one author and independently checked by a second author.

### Statistical analysis

Our study outcome was the prevalence of remission/LDA after TNFi discontinuation. We pooled induction-withdrawal studies and maintenance discontinuation studies separately, and for each treatment strategy, we pooled the outcomes of Disease Activity Score 28 (DAS28) < 3.2, DAS28 < 2.6, or Simplified Disease Activity Index (SDAI) ≤ 3.3 separately. For the few studies that reported the outcome as the proportion that did not restart biologic treatment, we conservatively classified these as DAS28 < 3.2. Since relapses are time-dependent and more likely with longer follow-up, we pooled results reported at 24–36 weeks after discontinuation and 37–52 weeks after discontinuation separately. We computed pooled prevalences using restricted maximum likelihood estimation random effects models with the double arcsine transformation, using the *metafor* package in R (version 4.2.2). We used *I*^2^ to assess heterogeneity among studies. For studies that also provided data on sustained remission/LDA in patients who continued TNFi treatment, we pooled these results and computed relative risks and risk differences of remission/LDA between discontinuation and continuation arms, using random effects models implemented in OpenMeta (www.cebm.brown.edu/openmeta).

We analyzed predictors at the time of TNFi discontinuation by comparing patients who maintained remission/LDA or not, based on the remission/LDA criterion in each study. For continuous predictors, we used mean values to compute standardized mean differences (SMD) between the groups and pooled the SMDs using DerSimonian and Laird random effects models in OpenMeta. SMDs represent the number of standard deviations by which two groups differ, with positive values indicating higher means in patients with sustained remission. For studies reporting medians, we used the methods of McGrath to estimate means [21]. For categorical predictors, we computed odds ratios for remission/LDA from reported proportions, or used the study’s reported odds ratios, and pooled these using random effects models in OpenMeta. If only hazard ratios were reported, we pooled these separately. We harmonized the direction of associations across studies so that successful discontinuation was the outcome.

In sensitivity analyses, we excluded studies rated as high risk of bias with the ROB2 tool, or serious or critical risk of bias with the ROBINS-I tool.

## Results

### Search results

**Fig. 1:**
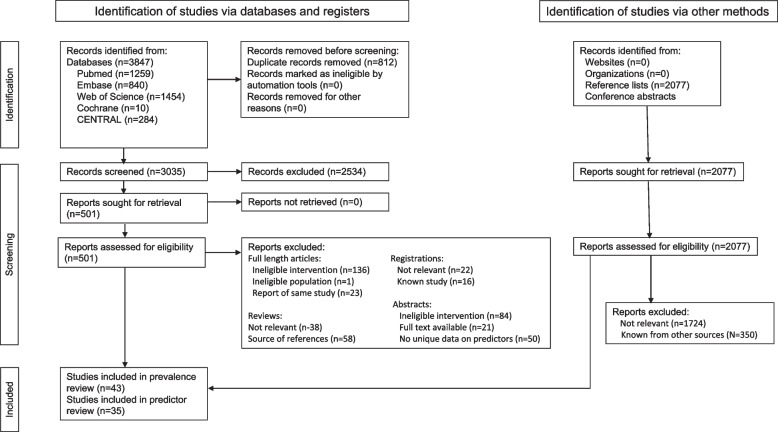
Flow diagram of study inclusion. Error bars represent 95% confidence intervals

Of 3035 unique articles identified in electronic searches and 2077 articles screened from secondary sources, we included 43 articles in the systematic review of the prevalence of sustained remission/LDA after discontinuation and 37 studies in the scoping review of predictors (Fig. 1). Of the 43 articles in the systematic review, 22 articles reported induction-withdrawal studies and 22 articles reported studies of maintenance TNFi discontinuation, with 1 article including both groups [22–64]. Data on predictors were reported in 12 induction-withdrawal articles [27, 33–36, 39, 41, 43, 65–68] and 22 maintenance discontinuation articles [44, 46, 47, 49–52, 54–56, 59–62, 64, 69–76].

### Sustained remission/LDA in induction-withdrawal studies

These studies included 5 double-blind controlled trials [22–26], 1 open-label trial [27], and 16 studies in which TNFi discontinuation was observational [28–43] (Table 1 and Supplemental Table 3).

**Table 1:** Proportion of patients with sustained remission or low disease activity in induction-withdrawal studies

Reference	Drug	RA duration	Activity at discontinuation	Number discontinued/ continued	Follow-up (weeks)	DAS28 < 3.2 at end (%)		DAS28 < 2.6 at end (%)		SDAI ≤ 3.3 at end (%)	
						Discontinued	Continued	Discontinued	Continued	Discontinued	Continued
**Double-blind controlled trials**											
Smolen OPTIMA 2014 [22]	ADA	Early	DAS28-CRP < 3.2 at weeks 22 and 26	102/105	52	81.2	91.4	66.3	85.7	50.5	61.9
Emery 2014 [23]	ETA	Early	DAS28-ESR < 3.2 at week 39 and DAS28-ESR < 2.6 at week 52	65/63	39	69.2	88.8	53.8	79.3	-	-
Smolen PRESERVE 2013 [24]	ETA	Est	DAS28-ESR < 3.2 over 24 weeks	200/202	52	42.6	82.6	29.4	66.7	11.7	37.8
Pavelka 2017 [25]	ETA	Est	DAS28-ESR < 3.2 at week 24	176/167	28	17	44	13	34	13	25
Weinblatt 2017 [26]	CTZ	Early	DAS28-ESR < 3.2 for 12 weeks	82/84	52	39.2	48.8	33.3	44.0	-	-
**Open-label trials**											
Yamanaka 2016 [27]	ETA	Early	DAS28 < 2.6 for 6 months	34/33	52	-	-	53.6	87.5	46.4	81.3
**Observational studies**											
Quinn 2005 [28]	INF	Early	DAS28 < 2.6	6/-	50	-	-	83	-	-	-
Van der Bijl 2007 [29]	INF	Early	DAS ≤ 2.4 for 6 months	77/-	66	-	-	87.0	-	-	-
Nawata 2008 [30]	INF	Early	DAS28-ESR < 2.6 for ≥ 6 months	9/-	24	-	-	100	-	-	-
Soubrier 2009 [31]	ADA	Early	DAS28 < 3.2 after 12 weeks of treatment	33/	40	33.3	-	-	-	-	-
Lagana 2009 [32]	ETA	Early	DAS < 1.6 after 12 months	8/-	52	-	-	100	-	-	-
Saleem 2010 [33]	ADA 74% INF 26%	Early	DAS28 < 2.6 for ≥ 6 months	27/-	96	-	-	59.2	-	-	-
Migliore 2010 [34]	ETA 43% ADA 28% INF 28%	Early	DAS28 < 3.2	21/-	24	61.9	-	-	-	-	-
Migliore 2011 [35]	ETA 46% ADA 26% INF 28%	Early	DAS28 < 3.2	50/-	52	42.0	-	-	-	-	-
Harigai 2012 [36]	ADA	Est	DAS28-CRP < 2.7	22/24	52	-	-	18.1	66.6	-	-
Nam IDEA 2014 [37]	INF	Early	DAS < 1.6 for 6 months	14/-	28	-	-	78.6	-	-	-
Nam EMPIRE 2014 [38]	ETA	Early	No tender or swollen joints for > 26 weeks	2/-	26	-	-	100	-	-	-
Tanaka HONOR 2015 [39]	ADA	Est	DAS28-ESR < 2.6 for ≥ 6 months	52/23	52	62	91	48	83	60	70
Smolen 2015 [40]	CTZ	Est	CDAI ≤ 2.8 for 5 weeks	17/6	28	-	-	-	-	17.6^b^	33.3^b^
Tanaka HOPEFUL-2 2016 [41]	ADA	Early	DAS28-ESR < 3.2 for ≥ 6 weeks	80/73	52	80.0	97.2	-	-	-	-
Inui 2018 [42]	ETA	Est	DAS28-ESR < 3.2 at one visit	18/-	96	27.7	-	-	-	-	-
Tanaka RRRR 2020 [43]	INF	Est	SDAI ≤ 3.3	119/-	52	63.8^a^	-	-	-	-	-

*RA* rheumatoid arthritis, *Est* established, *DAS28* Disease Activity Score 28, *SDAI* Simplified Disease Activity Index, *CDAI* Clinical Disease Activity Index, *ADA* adalimumab, *ETA* etanercept, *INF* infliximab, *CTZ* certolizumab, *GOL* golimumab, *ESR* erythrocyte sedimentation rate, *CRP* C-reactive protein

^a^Off biologics

^b^CDAI ≤ 2.8

The criterion for TNFi discontinuation was DAS28 < 3.2 in 9 studies, DAS28 < 2.6 in 9 studies, and other indicators in 4 studies. The number of patients who discontinued TNFi ranged from 2 to 200 (median 34; total 1183), with larger samples in the trials. Seven studies examined etanercept, 5 examined infliximab, 5 examined adalimumab, 2 examined certolizumab, and 3 examined various TNFi. Follow-up varied from 24 to 96 weeks. Thirteen studies reported results at 37–52 weeks after TNFi discontinuation, and 6 studies reported results at 24–36 weeks. The proportion of patients with sustained remission/LDA after TNFi discontinuation varied widely (Table 1).

#### Remission prevalence after discontinuation

**Fig. 2:**
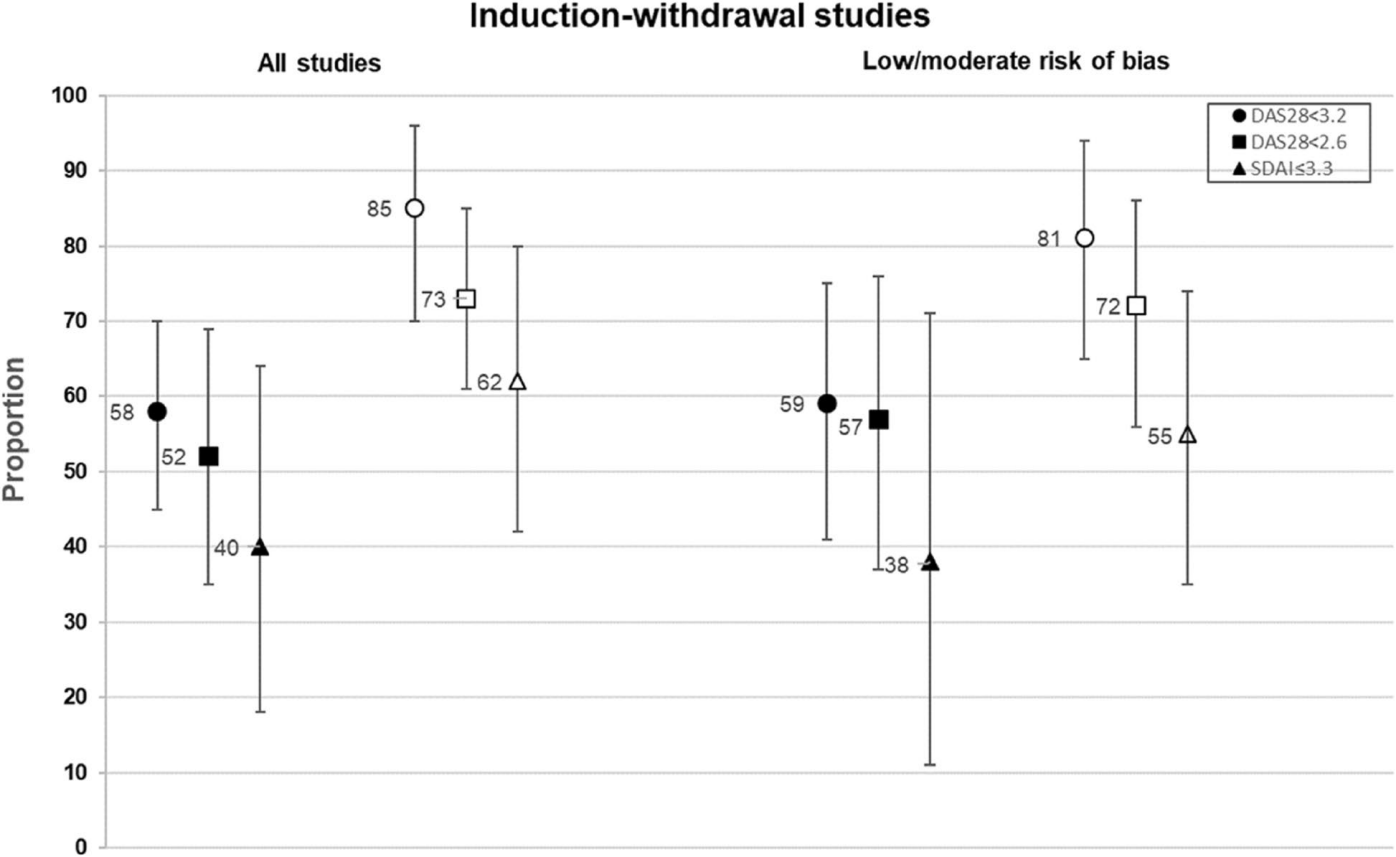
Pooled proportions having sustained remission/low disease activity at 37–52 weeks after either discontinuation or continuation of tumor necrosis factor inhibitor treatment in induction-withdrawal studies. Circles represent the DAS28 < 3.2 outcome, squares represent the DAS28 < 2.6 outcome, and triangles represent the SDAI ≤ 3.3 outcome. Closed symbols represent tumor necrosis factor inhibitor discontinuation arms, and open symbols represent continuation arms. Error bars represent 95% confidence intervals

In the pooled analysis, 58% had DAS28 < 3.2 and 52% had DAS28 < 2.6 at 37–52 weeks after discontinuation, with high heterogeneity among studies (Fig. 2 and Supplemental table 4).

Only four studies reported SDAI-based results, and 40% of patients had SDAI ≤ 3.3 after discontinuation. The proportion remaining in remission/LDA was therefore lower with more stringent definitions of remission. At 24–36 weeks after TNFi discontinuation, 36% of patients maintained DAS28 < 3.2, 73% had DAS28 < 2.6, and 12% had SDAI ≤ 3.3 (Supplemental table 4).

#### Sensitivity analysis and study heterogeneity

The double-blind controlled trials were rated as having a low or moderate risk of bias, while the open-label trial was rated as having a high risk of bias (Supplemental Fig. 1). Seven observational studies were judged as having a serious risk of bias (Supplemental Fig. 2). In the sensitivity analysis, pooled results were similar when only studies with low or moderate risk of bias were examined (Fig. 2 and Supplemental table 4).

We explored potential heterogeneity by disease activity, duration of RA, and study design among the 9 studies that reported DAS28 < 2.6 outcomes at 37–52 weeks. Among the six studies that required DAS28 < 2.6 at the time of discontinuation [23, 27, 28, 32, 36, 39], the proportion with DAS28 < 2.6 at follow-up 1 year later was 58% (95% CI 33, 82), compared to 42% (95% CI 20, 67) among the three studies that required DAS28 < 3.2 at TNFi discontinuation [22, 24, 26] (*p* = 0.42). Among the six studies in early RA [22, 23, 26–28, 32], the pooled proportion with DAS28 < 2.6 at follow-up was 63% (95% CI 42, 82), compared to 32% (95% CI 17, 49) in three studies in established RA [24, 36, 39] (*p* = 0.05). Among the five controlled trials [22–24, 26, 27], the pooled prevalence with DAS28 < 2.6 at follow-up was 47% (95% CI 30, 63), while among the four observational studies [28, 32, 36, 39], the pooled prevalence was 58% (95% CI 25, 88) (*p* = 0.84).

#### Retreatment

In five studies that reported on retreatment (64 patients combined) after relapse following TNFi discontinuation, 96% (95% CI 85, 100) regained remission/LDA after resuming TNFi treatment (Supplemental Table 3).

#### Remission prevalence with TNFi continuation in controlled studies

In the controlled studies, 85%, 73%, and 62% of patients who continued TNFi treatment maintained DAS28 < 3.2, DAS28 < 2.6, and SDAI ≤ 3.3, respectively, at 37–52 weeks’ follow-up (Fig. 2 and Supplemental table 4). In pooled analyses of controlled studies that compared those who discontinued TNFi to those who continued TNFi, the risk ratio of sustained DAS28 < 3.2 was 0.69, the risk ratio of sustained DAS28 < 2.6 was 0.58, and the risk ratio of sustained SDAI ≤ 3.3 was 0.59 (Supplemental table 5). Pooled risk differences were − 22.2%, − 27.3%, and − 18.4% for these outcomes, indicating that absolute relapses in the discontinuation group exceeded those in the paired continuation group by these amounts.

### Sustained remission/LDA after discontinuation of maintenance TNFi

These studies included 3 double-blind controlled trials [44–46], 2 open-label trials [47, 48], and 17 studies in which TNFi discontinuation was observational [33, 49–64], including 4 registry studies [54, 55, 62, 63] (Table 2 and Supplemental table 6).

**Table 2:** Proportions with sustained remission or low disease activity among studies of discontinuation of maintenance tumor necrosis factor inhibitor treatment

Reference	Drug	RA duration	Activity at Discontinuation	Number discontinued/continued	Follow-up (weeks)	DAS28 < 3.2 at end (%)		DAS28 < 2.6 at end (%)		SDAI ≤ 3.3 at end (%)	
						Discontinued	Continued	Discontinued	Continued	Discontinued	Continued
**Double-blind controlled trials**											
Van Vollenhoven 2016 [44]	ETA	Est	DAS28-ESR ≤ 3.2 for ≥ 11 months	23/23	48	13.0	52.1	-	-	-	-
Emery 2020 [45]	ADA	Est	DAS28-ESR < 2.6 for ≥ 6 months	20/102	36	-	-	57.8	62.7	-	-
Curtis 2021 [46]	ETA	Est	SDAI ≤ 3.3 for 24 weeks	101/51	48	-	-	-	-	28.7	52.9
**Open-label trials**											
Chatzidionysiou 2016 [47]	ADA	Est	DAS28 < 2.6 ≥ 3 months	15/16	28	-	-	33.3	93.7	-	-
Ghiti Moghadam 2016 [48]	ETA 40% ADA 51% INF 5%	Est	DAS28-ESR < 3.2 ≥ 6 months	531/286	52	48.8	81.8	29.7	56.9	-	-
**Observational studies**											
Brocq 2009 [49]	ETA 65% ADA 25% INF 10%	Est	DAS28 < 2.6 for ≥ 6 months	21/-	52	25.0	-	-	-	-	-
Tanaka RRR 2010 [50]	INF	Est	DAS28-ESR < 3.2 for ≥ 6 months	102/-	52	54.9	-	43.1	-	-	-
Saleem 2010 [33]	ETA 25% ADA 10% INF 65%	Est	DAS28 < 2.6 for ≥ 6 months	20/-	96	-	-	15.0	-	-	-
Iwamoto 2014 [51]	ETA 9% ADA 19% INF 53% GOL 15% CTZ 3%	Est	DAS28 < 2.6 (no duration specified)	32/-	24	59.3	-	-	-	-	-
Kurasawa 2014 [52]	INF	Est	DAS28-CRP < 2.6 for ≥ 6 months	31/-	52	-	-	55.0	-	-	-
Kadar 2014 [53]	TNFi	Est	Long-term remission	5/-	24	-	-	100	-	-	-
Kavanaugh 2015 [54]	TNFi	Est	CDAI ≤ 10 (no duration specified)	717/-	52	73.4^a^	-		-	-	-
Yoshida 2016 [55]	Biologics (82% TNFi)	Est	CDAI ≤ 2.8	46/-	52	32.6^a^	-	-	-	-	-
Kawashiri 2017 [56]	ETA 10% ADA 23% INF 47% GOL 13% CTZ 7%	Est	DAS28-ESR < 3.2 for ≥ 3 months	30/-	52	46.6^a^	-	-	-	-	-
Kimura 2019 [57]	ADA	Est	DAS28-ESR < 2.6 for ≥ 6 months	4/29	52	-	-	25.0	75.8	-	-
Ito 2019 [58]	ADA	Est	Sustained clinical remission	20/-	24	100	-	95	-	-	-
Naniwa 2020 [59]	ETA 28% ADA 16% INF 47% GOL 3% CTZ 5%	Early	SDAI ≤ 11, off corticosteroids for ≥ 6 months	95/-	52	-	-	66.2	-	63.8	-
Takai 2020 [60]	INF	Est	DAS28-ESR < 2.6, usually > 2 years	18/-	48	55.6	-	55.6	-	-	-
Kameda 2021 [61]	ETA 38% ADA 12% INF 23% GOL 19% CTZ 8%	Est	SDAI ≤ 3.3 for ≥ 3 months	26/-	52	46.1	-	-	-	-	-
Ochiai 2021 [62]	ETA 23% ADA 10% INF 67%	Est	DAS28-ESR < 3.2 (no duration reported)	39/-	52	56.4	-	-	-	-	-
Burkard 2021 [63]	ETA 28% ADA 40% INF 12% GOL 13% CTZ 7%	Est	DAS28 < 2.6 or RADAI < 1.5	212/-	104	30.7^a^	-	-	-	-	-
Nagatani 2021 [64]	ETA 50% ADA 12% INF 38%	Est	DAS28-CRP < 2.3 for ≥ 12 months	34/-	104	-	-	38.2	-	-	-

*RA* rheumatoid arthritis, *Est* established, *DAS28* Disease Activity Score 28, *SDAI* Simplified Disease Activity Index, *CDAI* Clinical Disease Activity Index, *RADAI* Rheumatoid Arthritis Disease Activity Index, *ADA* adalimumab, *ETA* etanercept, *INF* infliximab, *CTZ* certolizumab, *GOL* golimumab, *ESR* erythrocyte sedimentation rate, *CRP* C-reactive protein

^a^Off biologics, with or without CDAI remission

Six studies used DAS28 < 3.2 as the criterion for discontinuation, 9 studies used DAS28 < 2.6, 2 studies used SDAI ≤ 3.3, and 5 studies used other criteria. Thirteen studies included patients treated with different TNFi. Minimum durations of remission/LDA were 3 months in 3 studies, 6 months in 9 studies, longer than 6 months in 3 studies, and unspecified in 7 studies. The number of patients who discontinued TNFi ranged from 4 to 717 (median 30; total 2142). Five studies reported outcomes at 24–36 weeks, 14 studies reported results at 37–52 weeks, and 3 studies reported outcomes at longer times.

#### Remission prevalence after discontinuation

**Fig. 3:**
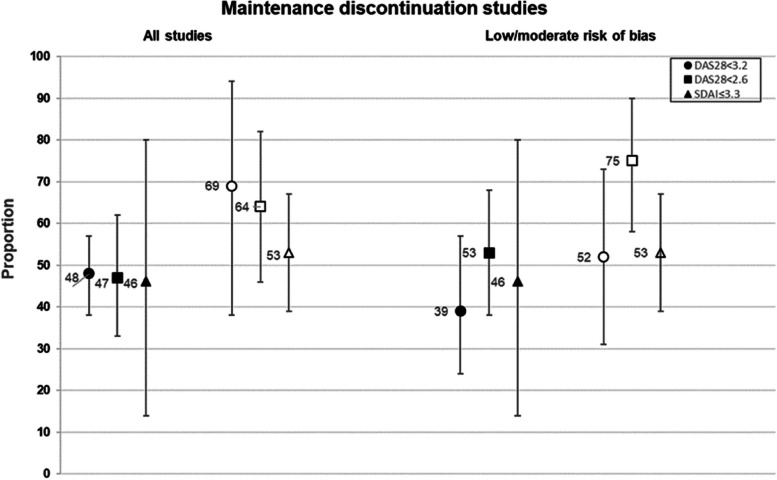
Pooled proportions having sustained remission/low disease activity at 37–52 weeks after either discontinuation or continuation of tumor necrosis factor inhibitor treatment in maintenance discontinuation studies. Circles represent the DAS28 < 3.2 outcome, squares represent the DAS28 < 2.6 outcome, and triangles represent the SDAI ≤ 3.3 outcome. Closed symbols represent tumor necrosis factor inhibitor discontinuation arms, and open symbols represent continuation arms

In the pooled results, 48% of patients had DAS28 < 3.2 at 37–52 weeks after discontinuation, 47% had DAS28 < 2.6, and 46% had SDAI ≤ 3.3, with high heterogeneity among studies (Fig. 3 and Supplemental table 7). At 24–36 weeks after TNFi discontinuation, 85% of patients maintained DAS28 < 3.2, and 75% had DAS28 < 2.6.

The blinded trials were rated as having a low or moderate risk of bias, while the open-label trials had high risk of bias (Supplemental Fig. 3). Nine observational studies were rated as having a low or moderate risk of bias (Supplemental Fig. 4).

#### Sensitivity analysis and study heterogeneity

In the sensitivity analysis, the proportions of patients with successful discontinuation among studies with low or moderate risk of bias were similar to, or somewhat lower than, the proportions among all studies (Fig. 3 and Supplemental table 7).

Examining heterogeneity by RA activity, DAS28 < 2.6 at 37–52 weeks after discontinuation was only slightly more common among studies that required DAS28 < 2.6 at enrollment [52, 57, 60] compared to studies that required DAS28 < 3.2 at enrollment [48, 50, 59] (53% (95% CI 38, 68) versus 45% (95% CI 25, 67)). All studies of maintenance treatment discontinuation examined patients with established RA. The proportion with DAS28 < 2.6 at follow-up was higher in the five observational studies [50, 52, 57, 59, 60] (53%; 95% CI 40, 66) than in the one clinical trial [48] (29%; 95% CI 25, 34) (*p* = 0.04).

#### Retreatment

Among 11 studies that reported on retreatment of relapses (360 patients combined), the pooled proportion of patients who regained remission was 86% (95% CI 71, 98) (Supplemental table 6).

#### Remission prevalence with TNFi continuation in controlled studies

Among patients in controlled studies who continued TNFi, 69% maintained DAS28 < 3.2, 64% maintained DAS28 < 2.6, and 53% maintained SDAI ≤ 3.3 at 37–52 weeks, although the number of studies was small (Fig. 3 and Supplemental table 7). In paired analyses of studies that reported both discontinuation and continuation arms, sustained remission/LDA was more likely among those who continued TNFi, with risk ratios that ranged from 0.47 to 0.57 (Supplemental table 8). Risk differences indicated that absolute rates of maintaining DAS28 < 3.2 were, on average, 33.4% lower with discontinuation, and of maintaining DAS28 < 2.6 were 32.1% lower with discontinuation.

### Predictors of successful discontinuation in induction-withdrawal studies

Collectively, data on 18 different predictors were reported (Table 3 and Supplemental table 9) [27, 33–36, 39, 41, 43, 65–68]. However, only 8 predictors were reported by more than 3 studies, and pooling was limited because studies used different effect size measures. Older age was not predictive in studies that reported mean ages, but older age groups were less likely to have successful discontinuation in two studies that reported odds ratios [43, 65]. Mean duration of RA was shorter among patients with successful discontinuation. Longer duration of TNFi treatment prior to discontinuation was associated with lower likelihood of success.

**Table 3:** Predictors of sustained remission in induction-withdrawal studies and studies of discontinuation of maintenance tumor necrosis factor inhibitor (TNFi) treatment

	Induction-withdrawal studies				Maintenance discontinuation studies			
Predictor	Number of studies	Effect size (SMD, OR, or HR)	*P*	*I*^2^	Number of studies	Effect size (SMD, OR, or HR)	*P*	*I*^2^
Older age, continuous, SMD	4	0.12 (− 0.56, 0.82)	0.72	71.6	11	− 0.15 (− 0.34, 0.03)	0.10	0
Older age, categorical, OR	2	0.58 (0.37, 0.89)	0.01	0	1	0.85 (0.57, 1.25)	0.83	-
Older age, categorical, HR	1	1.00 (0.98, 1.02)	0.99	-	2	1.03 (0.91, 1.17)	0.62	0
Women vs Men, OR	5	0.75 (0.49, 1.15)	0.19	0	11	0.91 (0.67, 1.25)	0.57	0
Women vs Men, HR	1	0.91 (0.50, 1.66)	0.97	-	2	0.98 (0.82, 1.19)	0.84	0
Duration of RA, continuous, SMD	4	− 0.40 (− 0.72, − 0.08)	0.02	4.9	11	− 0.26 (− 0.45, − 0.07)	0.006	0
Duration of RA, categorical, OR	2	0.76 (0.44, 1.31)	0.32	50.5	2	0.35 (0.13, 0.91)	0.03	63.0
Duration of RA, categorical, HR	1	0.78 (0.69, 0.88)	< 0.001	-	1	1.05 (0.74, 1.47)	0.77	-
Duration of TNFi treatment prior to discontinuation, continuous, SMD	2	− 0.45 (− 0.91, 0.01)	0.06	0	5	0.06 (− 0.66, 0.79)	0.86	73.4
Duration of TNFi treatment prior to discontinuation, categorical, OR	0	-	-	-	2	1.22 (0.95, 1.57)	0.12	57.9
Duration of TNFi treatment prior to discontinuation, categorical, HR	1	0.54 (0.41, 0.78)	0.002	-	0	-	-	-
Length of remission at time of TNFi discontinuation, continuous, SMD	0	-	-	-	6	0.13 (− 0.31, 0.57)	0.56	53.6
Time to reach remission with TNFi, continuous, SMD	0	-	-	-	3	− 0.36 (− 0.69, − 0.03)	0.03	0
Time to reach remission with TNFi, categorical, OR	1	4.66 (0.70, 31.03)	0.82	-	0	-	-	-
Time to reach remission with TNFi, categorical, HR	0	-	-	-	1	0.67 (0.47, 0.95)	0.03	-
Type of TNFi: Monoclonal antibody vs Etanercept, OR	0	-	-	-	6	1.64 (0.98, 2.74)	0.06	26.4
Methotrexate dose, continuous, SMD	2	− 0.18 (− 0.57, 0.20)	0.34	0	9	0.05 (− 0.14, 0.25)	0.57	0
Methotrexate dose, categorical, OR	1	0.78 (0.46, 1.34)	0.37	-	0	-	-	-
Glucocorticoid use, categorical, OR	2	1.30 (0.11, 15.17)	0.84	69.4	7	0.93 (0.46, 1.91)	0.85	23.6
Glucocorticoid use, categorical, HR	0	-	-	-	1	0.56 (0.29, 1.08)	0.09	-
RF value, continuous, SMD	3	0.03 (− 0.55, 0.61)	0.91	54.2	2	0.19 (− 0.26, 0.65)	0.40	25.6
RF positive vs negative, OR	2	0.73 (0.45, 1.20)	0.21	0	10	0.75 (0.54, 1.03)	0.08	0
RF positive vs negative, HR	1	0.83 (0.41, 1.67)	0.62	-	0	-	-	-
ACPA titer, continuous, SMD	1	− 0.27 (− 0.82, 0.27)	0.35	-	0	-	-	-
ACPA positive vs negative, OR	1	0.40 (0.11, 1.40)	0.16	-	9	0.86 (0.62, 1.20)	0.39	0
ACPA positive vs negative, HR	1	0.66 (0.34, 1.25)	0.22	-	0	-	-	-
HLA shared epitope present vs absent, HR	1	0.25 (0.09, 0.71)	0.008	-	0	-	-	-
Radiographic damage, Sharp Score continuous, SMD	1	− 0.47 (− 1.02, 0.07)	0.11	-	3	− 0.50 (− 0.82, − 0.17)	0.002	1.3
Radiographic damage, categorical, OR	2	1.25 (0.34, 4.59)	0.73	28.4	4	0.78 (0.66, 0.92)	0.004	2.3
Radiographic damage, categorical, HR	1	0.98 (0.96, 1.00)	0.05	-	0	-	-	-
BMI continuous, SMD	0	-	-	-	1	0.32 (− 0.09, 0.75)	0.14	-
BMI categorical, OR	0	-	-	-	2	0.71 (0.51, 0.99)	0.04	0
BMI categorical, HR	1	0.96 (0.89, 1.04)	0.69	-	1	0.80 (0.66, 0.96)	0.02	-
Smoker vs non-smoker, OR	0	-	-	-	1	0.63 (0.21, 1.88)	0.41	-
Smoker vs non-smoker, HR	1	0.41 (0.23, 0.71)	0.002	-	1	0.83 (0.70, 0.99)	0.04	-
HAQ continuous, SMD	4	− 0.33 (− 0.67, 0)	0.05	12.1	7	0 (− 0.25, 0.24)	0.99	0
HAQ categorical, OR	1	0.98 (0.60, 1.60)	0.94	-	0	-	-	-
HAQ categorical, HR	1	0.66 (0.34, 1.25)	0.22	-	1	0.82 (0.69, 0.97)	0.03	-
Disease activity, continuous, SMD	4	− 0.73 (− 1.30, − 0.16)	0.02	57.4	8	− 0.21 (− 0.59, 0.16)	0.26	51.7
Disease activity, categorical, OR	2	0.70 (0.37, 1.32)	0.27	69.0	3	0.47 (0.16, 1.38)	0.17	83.0
Disease activity, categorical, HR	2	0.71 (0.45, 1.12)	0.14	59.6	2	0.76 (0.65, 0.88)	< 0.01	0
MBDA > 44 vs ≤ 44, categorical, OR	1	0.08 (0.004, 1.67)	0.20	-	1	0.43 (0.24, 0.75)	0.003	-
Ultrasound Grey scale, continuous, SMD	1	− 0.45 (− 1.34, 0.43)	0.25	-	4	− 0.02 (− 0.36, 0.33)	0.93	0
Ultrasound power Doppler continuous, SMD	1	− 0.08 (− 0.96, 0.80)	0.97	-	4	− 0.30 (− 0.68, 0.07)	0.12	13.4
Ultrasound power Doppler categorical, HR	0	-	-	-	2	0.34 (0.09, 1.31)	0.12	70.1

*SMD* standardized mean difference, *OR* odds ratio, *HR* hazard ratio, *RA* rheumatoid arthritis, *RF* rheumatoid factor, *ACPA* anti-citrullinated protein antibody, *HLA* human leukocyte antigen, *BMI* body mass index, *HAQ* Health Assessment Questionnaire, *MBDA* Multi-biomarker disease activity score

Human leukocyte antigen (HLA) shared epitope, radiographic damage, and smoking were associated with a lower likelihood of successful discontinuation, based on one study [66]. Mean Health Assessment Questionnaire (HAQ) scores and mean disease activity scores were lower among patients with successful discontinuation. There were no associations with other predictors, including sex, seropositivity, and ultrasound measures. Serum matrix metalloproteinase-3 did not predict relapse in one study [39], while relapses were associated with lower proportions of peripheral blood naïve T cells and higher proportions of regulatory T cells in another study [33].

Few induction-withdrawal studies with low or moderate risk of bias reported on predictors (Supplemental table 10). Duration of RA was not clearly predictive in this subset.

### Predictors of successful discontinuation of maintenance TNFi treatment

More information was available among these studies, with data on 17 predictors reported in more than 3 studies (Table 3 and Supplemental table 9) [44, 46, 47, 49–52, 54–56, 59–62, 64, 69–75]. Mean duration of RA was shorter among patients with successful discontinuation, as was a shorter time to reach remission with TNFi treatment [55]. Patients treated with monoclonal TNFi tended to have more successful discontinuation than those receiving etanercept. Patients with more radiographic damage and obese patients were less likely to have successful discontinuation. Smoking, higher HAQ scores, and higher disease activity were associated with lower likelihoods of successful discontinuation only in two registry studies that reported hazard ratios [54, 55]. Higher multi-biomarker disease activity score was associated with lower odds of successful discontinuation in one study [69]. There were no associations with other variables, including length of remission, seropositivity, and ultrasound measures.

Selected laboratory biomarkers were examined in individual studies. Among 12 serum cytokines or cytokine receptors, lower levels of interleukin-2 and higher levels of soluble TNF receptor 1 at baseline predicted flare after treatment discontinuation in a small cohort [61]. Nagatani reported that relapse was associated with high serum interleukin-34, chemokine ligand-1, and interleukin-1β, and low serum interleukin-19 and interleukin-2 [64]. A low proportion of MerTK^+^CD206^+^ synovial tissue macrophages was strongly associated with the risk of flare after TNFi discontinuation [76].

Among studies with low or moderate risk of bias, successful discontinuation was less likely among patients with longer durations of RA and more radiographic damage, but was not associated with other clinical variables (Supplemental table 10).

## Discussion

Discontinuation of TNFi treatment in patients with well-controlled RA has the potential to improve care by simplifying regimens, decreasing treatment-related side effects, and reducing costs, but comes with the risk of increased RA activity. Knowing the absolute risk of relapse is needed to inform decision-making. Because these risks, and the associated strength of evidence, may differ between short-term TNFi treatment in an induction-withdrawal strategy and discontinuation of long-term maintenance TNFi treatment, it is important to examine these risks separately. Our pooled results indicated that 58% of patients had DAS28 < 3.2 and 52% had DAS28 < 2.6 at approximately 1 year after withdrawal of induction treatment. Comparable proportions were 48% and 47% after discontinuation of maintenance TNFi treatment. Few studies reported SDAI remission or results at 24–36 weeks.

Two previous systematic reviews that included 16 and 12 studies, respectively, reported successful discontinuation in 53% and 62% of patients [7, 77]. However, these reviews pooled studies that had different criteria for remission and different lengths of follow-up, and did not distinguish between the two clinical scenarios of discontinuation, limiting the specificity of their results. These results were comparable to our findings in induction-withdrawal studies, but were higher than our results for maintenance discontinuation studies. Successful discontinuation was more common in induction-withdrawal studies, which may reflect greater responsiveness in early RA. The proportion with successful discontinuation also decreased with increasing stringency of remission, particularly so for SDAI ≤ 3.3. Stratifying by the timing of responses is also important because more relapses would be expected with longer follow-up. In maintenance discontinuation studies, for example, DAS28 < 2.6 was maintained by 75% at 24–36 weeks but only 47% at 37–52 weeks. We did not observe a similar pattern in the induction-withdrawal studies, although few studies reported results at early times. These observations underscore differences by clinical scenario, outcome, and time.

Other reviews summarized discontinuation studies qualitatively [4, 8, 9, 12, 78–80] or included only controlled trials and focused on comparisons between discontinuation and continuation of TNFi [10, 11, 81–83]. In these meta-analyses, risk ratios for LDA with discontinuation ranged from 0.44 to 0.75, and risk ratios for DAS28 remission ranged from 0.45 to 0.71 [10, 80–82]. We focused on the absolute risks associated with discontinuation, because absolute frequencies of relapse are an important consideration in individual patient decision-making. Data on patients who continued TNFi treatment showed that, on average, 15% of patients did not maintain LDA and 27% did not maintain DAS28 remission for periods up to 1 year in induction studies, while 31% and 36% of patients who continued maintenance TNFi treatment similarly relapsed. These results provide useful context for interpreting the proportions in the discontinuation arms, highlighting that not all these relapses are necessarily attributable to TNFi discontinuation. Many would have been expected regardless of TNFi discontinuation. Risk differences assess this directly and indicate that relapses attributable to discontinuation ranged from 20 to 33%.

It is important to note that there was substantial heterogeneity among studies, even with the same design, outcome, and length of follow-up. This may be due to differences in inclusion criteria, patient selection, and depth of remission. That 15–47% of patients lose remission over 1 year despite continuing on TNFi treatment may be due to the limited specificity of these remission criteria, but also indicates that remission in RA does not indicate a cure.

Among induction-withdrawal studies, TNFi discontinuation was more successful in patients with early RA, approaching the prevalence seen in those who continued TNFi (63% versus 73%). Greater success in early RA and among patients with deeper remission has been suggested previously [13, 14, 78, 84]. In our pooled analysis, associations with a shorter duration of RA and lower disease activity were also supported by multiple studies, as were lower HAQ scores and shorter duration of TNFi treatment. RA activity and HAQ were not found to be associated with successful discontinuation in studies that dichotomized these measures, perhaps due to reduced statistical power. Age, sex, seropositivity, and methotrexate dose were not predictive of successful discontinuation in induction-withdrawal studies. There were few data on other predictors.

Among studies of maintenance TNFi treatment, discontinuation was more successful among patients with shorter RA durations and less radiographic damage, as identified previously [14]. Given that radiographic changes are cumulative, it is not clear if radiographic damage predicts the risk of relapse independent of RA duration. Shorter time to remission with TNFi treatment was also associated with successful discontinuation. Interestingly, monoclonal TNFi tended to have more successful discontinuation than etanercept. Whether this is related to patient selection or different immunological effects is unclear. We found no association with other clinical variables, including disease activity, in contrast to induction-withdrawal studies [14].

Few studies examined immunological biomarkers, and it is difficult to draw conclusions about prognostic importance based on single studies. Given the general absence of clinical predictors, it may be that immunological markers will be key to identifying which patients will be able to maintain remission after TNFi discontinuation. Although subclinical joint inflammation is common in clinical remission [85], our results did not support the prognostic value of ultrasound in studies of TNFi discontinuation. Power Doppler positivity in remission has been associated with higher odds of relapse in one study, but this study did not examine treatment discontinuation [85]. In three studies of biologic tapering, ultrasound abnormalities predicted relapse, indicating that further evaluation of the potential prognostic value of ultrasound is warranted [86–88]. Subclinical joint inflammation by magnetic resonance imaging (MRI) has also been observed in many patients in clinical remission, but MRI has not been found to predict relapses on biologic tapering [45, 88, 89]. We did not identify prognostic studies of MRI in the setting of TNFi discontinuation.

Our study is limited by the definitions of remission used in the primary studies, which may be considered too liberal. Few studies used SDAI remission as either the inclusion criterion or outcome, and none used American College of Rheumatology Boolean criteria. Interestingly, the more stringent SDAI criterion resulted in both lower proportions of remission and higher proportions of relapses, reflecting increased difficulty of maintaining this level of RA activity over time. We focused on TNFi discontinuation, given there are few discontinuation studies of other biologics or csDMARDs, or of tapering, and pooling results of different strategies or medications would decrease the specificity of any conclusions. We included both observational studies and controlled trials. Although several studies were judged to have a high risk of bias, results were generally similar after excluding such studies. Pooling of results in the predictor analysis was limited by the diversity of effect measures in the primary studies. We cannot exclude the possibility of publication bias, which is difficult to identify in the presence of heterogeneity [90]. We tried to minimize publication bias by using a comprehensive search strategy that included trial registrations, abstracts, and no language restrictions. We also included articles whose main objective was not to determine the prevalence of remission after TNFi discontinuation.

## Conclusions

This study is the first to examine the outcomes of TNFi discontinuation separately in induction treatment and maintenance treatment. Almost one-half of patients were able to discontinue maintenance TNFi treatment and remain in remission for up to 1 year. More patients had successful discontinuation in induction-withdrawal studies, underscoring the differences in outcomes between these scenarios. In both cases, patients with early RA were more likely to have successful discontinuation. After induction treatment with TNFi, approximately 6 in 10 patients with early RA would remain in remission for up to 1 year after discontinuation, but only 3 in 10 patients with established RA would do so. After discontinuation of maintenance TNFi treatment, approximately 5 in 10 patients would remain in remission for up to 1 year. These results may be useful in shared decision-making with patients who are contemplating treatment de-escalation. More research is needed to identify how risks of relapse vary in patient subgroups.

### Supplementary Information

**Additional file 1: Supplemental table 1.** Database search terms. **Supplemental table 2.**  Studies commonly cited but excluded from this review. **Supplemental table 3.**  Detailed characteristics of induction withdrawal studies. **Supplemental table 4.**  Pooled proportions of patients having sustained remission/low disease activity after either discontinuation or continuation of tumor necrosis factor inhibitor (TNFi) treatment in induction-withdrawal studies, stratified by length of follow-up. **Supplemental figure 1.**  Risk of bias evaluation of controlled trials among induction-withdrawal studies of tumor necrosis factor inhibitors using the Risk of Bias-2 tool. **Supplemental figure 2.**  Risk of bias evaluation of observational studies of induction-withdrawal of tumor necrosis factor inhibitor treatment using the Risk of Bias in Non-randomised Studies of Interventions (ROBINS-I) tool. **Supplemental table 5.**  Relative risks and risk differences of sustained remission/low disease activity with discontinuation versus continuation of tumor necrosis factor inhibitor treatment in induction-withdrawal studies that reported both arms. **Supplemental table 6.**  Detailed characteristics of studies of discontinuation of maintenance treatment with tumor necrosis factor inhibitors. **Supplemental table 7.**  Pooled proportions of patients having sustained remission/low disease activity after either discontinuation or continuation of maintenance treatment with tumor necrosis factor inhibitor (TNFi), stratified by length of follow-up. **Supplemental figure 3.**  Risk of bias evaluation of controlled trials of discontinuation of maintenance treatment with tumor necrosis factor inhibitors using the Risk of Bias-2 tool. **Supplemental figure 4.**  Risk of bias evaluation in observational studies of discontinuation of maintenance treatment with tumor necrosis factor inhibitors, using the Risk of Bias In Non-randomised Studies of Interventions (ROBINS-1) tool. **Supplemental table 8.**  Relative risks and risk differences of sustained remission or low disease activity with discontinuation versus continuation of tumor necrosis factor inhibitor treatment in maintenance discontinuation studies that reported both arms. **Supplemental table 9.**  Predictors of successful discontinuation, by study. **Supplemental table 10.**  Predictors of sustained remission in studies of discontinuation of tumor necrosis factor inhibitor treatment among studies of low or moderate risk of bias. PRISMA checklist.

## Data Availability

All data were obtained from publicly available materials, and all data are included in the article or Supplement.

## Abbreviations

ACPA: Anti-citrullinated protein antibody
ADA: Adalimumab
BMI: Body mass index
CDAI: Clinical disease activity index
CI: Confidence interval
CRP: C-reactive protein
CTZ: Certolizumab
csDMARDs: Conventional synthetic disease modifying anti-rheumatic drugs
DAS28: Disease activity score 28
ESR: Erythrocyte sedimentation rate
ETA: Etanercept
GOL: Golimumab
HAQ: Health assessment questionnaire
HLA: Human leukocyte antigen
HR: Hazard ratio
INF: Infliximab
LDA: Low disease activity
MBDA: Multi-biomarker disease activity score
OR: Odds ratio
PICO: Population intervention comparator outcome
RA: Rheumatoid arthritis
RADAI: Rheumatoid arthritis disease activity index
RF: Rheumatoid factor
ROB2: Risk of bias 2
ROBINS-I: Risk of bias in non-randomized studies of interventions
SDAI: Simplified disease activity index
SMD: Standardized mean difference
TNFi: Tumor necrosis factor inhibitors
