# Supplementary material for: Prevalence and predictors of sustained remission/low disease activity after discontinuation of induction or maintenance treatment with tumor necrosis factor inhibitors in rheumatoid arthritis: a systematic and scoping review
Source: Arthritis Res Ther. 2023 Nov 20;25:222. doi: 10.1186/s13075-023-03199-0 (PMC10659063; doi:10.1186/s13075-023-03199-0)
Supplement: Supplementary file 1 — Additional file 1: Supplemental table 1. Database search terms. Supplemental table 2. Studies commonly cited but excluded from this review. Supplemental table 3. Detailed characteristics of induction withdrawal studies. Supplemental table 4. Pooled proportions of patients having sustained remission/low disease activity after either discontinuation or continuation of tumor necrosis factor inhibitor (TNFi) treatment in induction-withdrawal studies, stratified by length of follow-up. Supplemental figure 1. Risk of bias evaluation of controlled trials among induction-withdrawal studies of tumor necrosis factor inhibitors using the Risk of Bias-2 tool. Supplemental figure 2. Risk of bias evaluation of observational studies of induction-withdrawal of tumor necrosis factor inhibitor treatment using the Risk of Bias in Non-randomised Studies of Interventions (ROBINS-I) tool. Supplemental table 5. Relative risks and risk differences of sustained remission/low disease activity with discontinuation versus continuation of tumor necrosis factor inhibitor treatment in induction-withdrawal studies that reported both arms. Supplemental table 6. Detailed characteristics of studies of discontinuation of maintenance treatment with tumor necrosis factor inhibitors. Supplemental table 7. Pooled proportions of patients having sustained remission/low disease activity after either discontinuation or continuation of maintenance treatment with tumor necrosis factor inhibitor (TNFi), stratified by length of follow-up. Supplemental figure 3. Risk of bias evaluation of controlled trials of discontinuation of maintenance treatment with tumor necrosis factor inhibitors using the Risk of Bias-2 tool. Supplemental figure 4. Risk of bias evaluation in observational studies of discontinuation of maintenance treatment with tumor necrosis factor inhibitors, using the Risk of Bias In Non-randomised Studies of Interventions (ROBINS-1) tool. Supplemental table 8. Relative risks and risk differences of sus [file 13075_2023_3199_MOESM1_ESM.docx]

SUPPLEMENT

Supplemental table 1. Database search terms.

Supplemental table 2. Studies commonly cited but excluded from this review.

Supplemental table 3. Detailed characteristics of induction-withdrawal studies.

Supplemental table 4. Pooled proportions of patients having sustained remission/low disease activity after either discontinuation or continuation of tumor necrosis factor inhibitor (TNFi) treatment in induction-withdrawal studies, stratified by length of follow-up.

Supplemental figure 1. Risk of bias evaluation of controlled trials among induction-withdrawal studies of tumor necrosis factor inhibitors using the Risk of Bias-2 tool.

Supplemental figure 2. Risk of bias evaluation of observational studies of induction-withdrawal of tumor necrosis factor inhibitor treatment using the Risk of Bias in Non-randomised Studies of Interventions (ROBINS-I) tool.

Supplemental table 5. Relative risks and risk differences of sustained remission/low disease activity with discontinuation versus continuation of tumor necrosis factor inhibitor treatment in induction-withdrawal studies that reported both arms.

Supplemental table 6. Detailed characteristics of studies of discontinuation of maintenance treatment with tumor necrosis factor inhibitors.

Supplemental table 7. Pooled proportions of patients having sustained remission/low disease activity after either discontinuation or continuation of maintenance treatment with tumor necrosis factor inhibitor (TNFi), stratified by length of follow-up.

Supplemental figure 3. Risk of bias evaluation of controlled trials of discontinuation of maintenance treatment with tumor necrosis factor inhibitors using the Risk of Bias-2 tool.

Supplemental figure 4. Risk of bias evaluation in observational studies of discontinuation of maintenance treatment with tumor necrosis factor inhibitors, using the Risk of Bias In Non-randomised Studies of Interventions (ROBINS-1) tool.

Supplemental table 8. Relative risks and risk differences of sustained remission or low disease activity with discontinuation versus continuation of tumor necrosis factor inhibitor treatment in maintenance discontinuation studies that reported both arms.

Supplemental table 9. Predictors of successful discontinuation, by study.

Supplemental table 10. Predictors of sustained remission in studies of discontinuation of tumor necrosis factor inhibitor treatment among studies of low or moderate risk of bias.

PRISMA checklist.

Supplemental table 1. Database search terms.

**Pubmed**

((("arthritis, rheumatoid"[MeSH Terms] OR "rheumatoid arthritis"[Title/Abstract]) AND ("tumor necrosis factor inhibitor"[Title/Abstract] OR "biological"[Title/Abstract] OR "etanercept"[Title/Abstract] OR "infliximab"[Title/Abstract] OR "adalimumab"[Title/Abstract] OR "certolizumab"[Title/Abstract] OR "golimumab"[Title/Abstract]) AND ("remission"[Title/Abstract] OR "low disease activity"[Title/Abstract])) NOT ("editorial"[Publication Type] OR "letter"[Publication Type] OR "news"[Publication Type] OR "retraction of publication"[Publication Type])) AND 2005/01/01:2022/05/01[Date - Publication]

**Embase**

('rheumatoid arthritis'/exp OR 'rheumatoid arthritis' OR 'rheumatoid arthritis'/exp/mj) AND ('tumor necrosis factor inhibitor':ab,ti OR 'etanercept':ab,ti OR 'infliximab':ab,ti OR 'adalimumab':ab,ti OR 'certolizumab':ab,ti OR 'golimumab':ab,ti OR 'biologic':ab,ti) AND ('discontinuation':ti,ab,kw OR 'withdrawal':ti,ab,kw) AND ('remission':ti,ab,kw OR 'low disease activity':ti,ab,kw) Date 2005-2022 and pub type (article or conference abstract or review)

**Web of Science**

Rheumatoid arthritis(topic) AND tumor necrosis factor inhibitor(abs) OR etanercept(abs) OR infliximab(abs) OR adalimumab(abs) OR certolizumab(abs) OR golimumab(abs) OR biologic(abs) AND remission(abs) Date 2005-2022 and pub type (article or review)

**Cochrane Reviews**

(rheumatoid arthritis):ti,ab,kw AND ("tumor necrosis factor" OR "etanercept" OR "infliximab" OR "adalimumab" OR "certolizumab" OR "golimumab"):ti,ab,kw AND ("remission"):ti,ab,kw with Cochrane Library publication date Between Jan 2005 and May 2022 (Word variations have been searched)

**CENTRAL**

rheumatoid arthritis in Title Abstract Keyword AND "tumor necrosis factor" OR "etanercept" OR "infliximab" OR "adalimumab" OR "certolizumab" OR "golimumab" in Title Abstract Keyword AND "remission" in Title Abstract Keyword AND "discontinuation" OR "withdrawal" in Title Abstract Keyword - with Cochrane Library publication date Between Jan 2005 and May 2022 (Word variations have been searched)

Supplemental table 2. Studies commonly cited but excluded from this review.

**Tapering**

van Herwaarden N, van der Maas A, Minten MJ, van den Hoogen FH, Kievit W, van Vollenhoven RF, Bijlsma JW, van den Bemt BJ, den Broeder AA. Disease activity guided dose reduction and withdrawal of adalimumab or etanercept compared with usual care in rheumatoid arthritis: open label, randomised controlled, non-inferiority trial. BMJ 2015 Apr 9;350.

Fautrel B, Pham T, Alfaiate T, Gandjbakhch F, Foltz V, Morel J, Dernis E, Gaudin P, Brocq O, Solau-Gervais E, Berthelot JM. Step-down strategy of spacing TNF-blocker injections for established rheumatoid arthritis in remission: results of the multicentre non-inferiority randomised open-label controlled trial (STRASS: Spacing of TNF-blocker injections in Rheumatoid ArthritiS Study). Ann Rheum Dis 2016;75(1):59-67.

van Mulligen E, de Jong PH, Kuijper TM, van der Ven M, Appels C, Bijkerk C, Harbers JB, de Man Y, Molenaar TE, Tchetverikov I, Goekoop-Ruiterman YP. Gradual tapering TNF inhibitors versus conventional synthetic DMARDs after achieving controlled disease in patients with rheumatoid arthritis: first-year results of the randomised controlled TARA study. Ann Rheum Dis 2019;78(6):746-53.

van der Maas A, Kievit W, van den Bemt BJ, van den Hoogen FH, van Riel PL, den Broeder AA. Down-titration and discontinuation of infliximab in rheumatoid arthritis patients with stable low disease activity and stable treatment: an observational cohort study. Ann Rheum Dis 2012;71(11):1849-54.

Alivernini S, Peluso G, Fedele AL, Tolusso B, Gremese E, Ferraccioli G. Tapering and discontinuation of TNF-α blockers without disease relapse using ultrasonography as a tool to identify patients with rheumatoid arthritis in clinical and histological remission. Arthritis Res Ther 2016;18(1):1-7.

Vittecoq O, Desouches S, Kozyreff M, Nicolau J, Pouplin S, Rottenberg P, Sens N, Lequerre T, Avenel G. Relapse in rheumatoid arthritis patients undergoing dose reduction and withdrawal of biologics: are predictable factors more relevant than predictive parameters? An observational prospective real-life study. BMJ Open. 2019 Dec 18;9(12):e031467.

Brahe CH, Krabbe S, Østergaard M, Ørnbjerg L, Glinatsi D, Røgind H, Jensen HS, Hansen A, Nørregaard J, Jacobsen S, Terslev L, Huynh TK, Jensen DV, Manilo N, Asmussen K, Brown Frandsen P, Boesen M, Rastiemadabadi Z, Morsel Carlsen L, Møller JM, Krogh NS, Hetland ML. Dose tapering and discontinuation of biological therapy in rheumatoid arthritis patients in routine care - 2-year outcomes and predictors. Rheumatology (Oxford). 2019 Jan 1;58(1):110-119.

Naredo E, Valor L, De la Torre I, Montoro M, Bello N, Martínez-Barrio J, Martínez-Estupiñán L, Nieto JC, Ovalles-Bonilla JG, Hernández-Flórez D, González CM. Predictive value of Doppler ultrasound-detected synovitis in relation to failed tapering of biologic therapy in patients with rheumatoid arthritis. Rheumatology 2015;54(8):1408-14.

El Miedany Y, El Gaafary M, Youssef S, Ahmed I, Bahlas S, Hegazi M, Nasr A. Optimizing therapy in inflammatory arthritis: prediction of relapse after tapering or stopping treatment for rheumatoid arthritis patients achieving clinical and radiological remission. Clin Rheumatol 2016; 35:2915-23.

**DMARD discontinuation**

Haschka J, Englbrecht M, Hueber AJ, Manger B, Kleyer A, Reiser M, Finzel S, Tony HP, Kleinert S, Feuchtenberger M, Fleck M. Relapse rates in patients with rheumatoid arthritis in stable remission tapering or stopping antirheumatic therapy: interim results from the prospective randomised controlled RETRO study. Ann Rheum Dis 2016;75(1):45-51.

**Additional Reports on Included Cohorts**

Kavanaugh A, Fleischmann RM, Emery P, Kupper H, Redden L, Guerette B, Santra S, Smolen JS. Clinical, functional and radiographic consequences of achieving stable low disease activity and remission with adalimumab plus methotrexate or methotrexate alone in early rheumatoid arthritis: 26-week results from the randomised, controlled OPTIMA study. Ann Rheum Dis 2013;72(1):64-71.

Hirata S, Saito K, Kubo S, Fukuyo S, Mizuno Y, Iwata S, Nawata M, Sawamukai N, Nakano K, Yamaoka K, Tanaka Y. Discontinuation of adalimumab after attaining disease activity score 28-erythrocyte sedimentation rate remission in patients with rheumatoid arthritis (HONOR study): an observational study. Arthritis Res Ther 2013;15(5):1-8.

Van Der Kooij SM, Goekoop-Ruiterman YP, De Vries-Bouwstra JK, Güler-Yüksel M, Zwinderman AH, Kerstens PJ, Van Der Lubbe PA, De Beus WM, Grillet BA, Ronday HK, Huizinga TW. Drug-free remission, functioning and radiographic damage after 4 years of response-driven treatment in patients with recent-onset rheumatoid arthritis. Ann Rheum Dis 2009;68(6):914-21.

Klarenbeek NB, Güler-Yüksel M, van der Kooij SM, Han KH, Ronday HK, Kerstens PJ, Seys PE, Huizinga TW, Dijkmans BA, Allaart CF. The impact of four dynamic, goal-steered treatment strategies on the 5-year outcomes of rheumatoid arthritis patients in the BeSt study. Ann Rheum Dis 2011; 70(6):1039-46.

Goekoop‐Ruiterman YD, de Vries‐Bouwstra JK, Allaart CF, Van Zeben D, Kerstens PJ, Hazes JM, Zwinderman AH, Ronday HK, Han KH, Westedt ML, Gerards AH. Clinical and radiographic outcomes of four different treatment strategies in patients with early rheumatoid arthritis (the BeSt study): a randomized, controlled trial. Arthritis Rheum 2005;52(11):3381-90.

Goekoop-Ruiterman YP, de Vries-Bouwstra JK, Allaart CF, van Zeben D, Kerstens PJ, Hazes JM, Zwinderman AH, Peeters AJ, de Jonge-Bok JM, Mallée C, de Beus WM. Comparison of treatment strategies in early rheumatoid arthritis: a randomized trial. Ann Inter Med 2007;146(6):406-15.

Allart CF, Lems WF, Huizinga TWJ. The Best way of withdrawing biologic agents. Clin Exp Rheumatol 2013; 31 (Suppl 78); S14-S18.

Bejarano V, Conaghan PG, Quinn MA, Saleem B, Emery P. Benefits 8 years after a remission induction regime with an infliximab and methotrexate combination in early rheumatoid arthritis. Rheumatology 2010;49(10):1971-4.

Bassiouni H, Spargo CE, Vlahos B, Jones HE, Pedersen R, Shirazy K. Maintenance of remission with etanercept-DMARD combination therapy compared with DMARDs alone in African and Middle Eastern patients with active rheumatoid arthritis. Rheumatol Ther 2018; 5:149-58.

Wiland P, Dudler J, Veale D, Tahir H, Pedersen R, Bukowski J, Vlahos B, Williams T, Gaylord S, Kotak S. The Effect of Reduced or Withdrawn Etanercept-methotrexate Therapy on Patient-reported Outcomes in Patients with Early Rheumatoid Arthritis. J Rheumatol. 2016 Jul;43(7):1268-77.

**Per Protocol Discontinuation of Treatment, Not related to Remission**

Buch MH, Marzo-Ortega H, Bingham SJ, Emery P. Long-term treatment of rheumatoid arthritis with tumour necrosis factor alpha blockade: outcome of ceasing and restarting biologicals. Rheumatology (Oxford). 2004 Feb;43(2):243-4.

Detert J, Bastian H, Listing J, Weiß A, Wassenberg S, Liebhaber A, Rockwitz K, Alten R, Krüger K, Rau R, Simon C. Induction therapy with adalimumab plus methotrexate for 24 weeks followed by methotrexate monotherapy up to week 48 versus methotrexate therapy alone for DMARD-naive patients with early rheumatoid arthritis: HIT HARD, an investigator-initiated study. Ann Rheum Dis 2013;72(6):844-50.

Hashimoto M, Furu M, Yamamoto W, Fujimura T, Hara R, Katayama M, Ohnishi A, Akashi K, Yoshida S, Nagai K, Son Y. Factors associated with the achievement of biological disease-modifying antirheumatic drug-free remission in rheumatoid arthritis: the ANSWER cohort study. Arthritis Res Ther 2018;20(1):1-10.

Hørslev-Petersen K, Hetland ML, Ørnbjerg LM, Junker P, Pødenphant J, Ellingsen T, Ahlquist P, Lindegaard H, Linauskas A, Schlemmer A, Dam MY, Hansen I, Lottenburger T, Ammitzbøll CG, Jørgensen A, Krintel SB, Raun J, Johansen JS, Østergaard M, Stengaard-Pedersen K; OPERA Study-Group. Clinical and radiographic outcome of a treat-to-target strategy using methotrexate and intra-articular glucocorticoids with or without adalimumab induction: a 2-year investigator-initiated, double-blinded, randomised, controlled trial (OPERA). Ann Rheum Dis. 2016 Sep;75(9):1645-53.

Supplemental table 3. Detailed characteristics of induction-withdrawal studies.*

| Reference | Drug | Patients | Design | Remission criterion for discontinuation | Primary endpoint | Retreatment after relapse |
| --- | --- | --- | --- | --- | --- | --- |
| Smolen, Lancet 2014; 383:321-32 (OPTIMA) [22] | ADA | Early RA (< 12 months), TNFi naïve, MTX naïve | Active RA at entry; induction treatment with ADA/MTX (n =515) v. Placebo/MTX (n =517) for 26 weeks.  Those in ADA/MTX group with DAS28-CRP < 3.2 at week 22 and 26 (n = 207) randomized (double blind) to continue ADA (n=105) or discontinue ADA (eg Placebo group) (n=102) | DAS28-CRP < 3.2 | DAS28-CRP < 3.2 and change in total Sharp score <0.5 at week 78:  ADA group 70% versus discontinue group 58% | Not reported |
| Emery, N Engl J Med 2014; 371:1781-92  (PRIZE) [23] | ETA | Early RA (< 12 months), biologic naïve, MTX naïve | Active RA at entry; induction treatment in all with ETA/MTX for 52 wks.  Those with DAS28-ESR ≤ 3.2 at week 39 and DAS28-ESR < 2.6 at week 52 randomized (double blind) to  ETA 25mg/MTX (n=63)  Placebo/MTX (n=65)  Placebo/PLB (n=65) | DAS28-ESR < 3.2 at week 39 and <2.6 at week 52 | DAS28-ESR < 2.6 at week 76 and 91, and no glucocorticoid use in first 12 weeks:  ETA/MTX group 63%  Placebo/MTX 40%  Placebo/Placebo23% | Not reported |
| Smolen, Lancet 2013; 38:918-29  (PRESERVE) [24] | ETA | Established RA, biologic naïve | Active RA at entry (moderate) despite MTX; induction treatment with ETA/MTX for 36 weeks.  Those with mean DAS28-ESR ≤ 3.2 at week 12 to 36, and DAS28-ESR ≤ 3.2 at week 36 randomized (double blind) to  ETA 50/MTX (n=202), ETA 25/MTX (n=202),  Placebo/MTX (n=200) | DAS28-ESR ≤3.2 at week 36 | DAS28-ESR ≤ 3.2 at week 88 (i.e. 52 weeks after randomization):  Full dose group 82.6%  Reduced dose group 79.1%  Placebo group 42.6% | Not reported |
| Pavelka, Rheum Int 2017; 37:3749-57 [25] | ETA | Established RA | Active RA despite MTX; induction treatment with ETA/MTX for 24 weeks.  Those with DAS28-ESR < 3.2 at week 24 randomized (double blind) to continue ETA (n=167) or receive placebo (n=176) | DAS28-ESR < 3.2 | DAS28-ESR < 3.2 at week 52:  ETA group 44% (71/163) v Placebo group 17% (29/168)  Median time to flare after discontinuation= 84 d | Not reported |
| Weinblatt, Arthritis Rheum 2017; 69:1937-48  (C-EARLY) [26] | CTZ | Early RA (<12 months), DMARD naïve, seropositive | Active RA at entry; induction treatment with CTZ/MTX for 52 weeks.  Those with DAS28-ESR ≤ 3.2 at week 40 and week 52 randomized (double blind) to continue  full dose CTZ (n=84), reduced frequency CTZ (n=127), or Placebo (n=82). Followed for another 52 weeks. | DAS28-ESR ≤ 3.2 at week 40 and 52 | DAS28-ESR < 3.2 on all 5 follow-up visits through week 104 without flares:  Full dose group 48.8%,  Reduced frequency group 53.2%,  Placebo group 39.2% (p= 0.11 full dose vs placebo) | 8/10 pts who stopped CTZ and flared and were retreated with CTZ regained low disease activity within 12 weeks |
| Yamanaka, Mod Rheumatol 2016; 26:651-61  (ENCOURAGE) [27] | ETA | Mostly early RA (mean ≤ 2 years) | Moderately active RA despite MTX, randomized to ETA/MTX vs MTX open label for 12 months.  Those in ETA/MTX group with DAS28 < 2.6 at 6 and 12 months randomized to continue ETA (n=49) or discontinue ETA (n=50) (open label), followed for another 12 mo. | DAS28 < 2.6 at week 52.  (also structural remission and functional remission) | Only completer analysis reported (n=33 in continue ETA group; n=34 in discontinue group):  Continue group 87.5%, Discontinue group 53.6% (p < .01) | Not reported |
| Quinn, Arthritis Rheum 2005; 52:27-35 [28] | INF | Early RA (< 12 months), poor prognostic factors, DMARD naïve | Active RA randomized to INF/MTX (n=10) or Placebo/MTX (n=10) for 1 yr. INF stopped in all and observed for another 52 weeks. | DAS28 < 2.6 | MRI synovitis at week14.  Of 6 patients in DAS28 remission at time of INF stop, 5 maintained remission to week 104 | Not reported |
| Van der Bijl, AR 2007; 56:2129-34  (BEST) [29] | INF | Early RA (< 2 years), DMARD naïve | Active RA randomized to INF/MTX.  INF discontinued in patients with DAS ≤ 2.4 for 6 or more months (n=77)  Median of 9.9 months of INF; MTX tapering allowed during follow-up | DAS ≤ 2.4 for 6 months or longer | 67/77 (87.0%) off INF through year 2 with DAS ≤ 2.4 | 8/10 (80%) regained remission after reintroduction of INF |
| Nawata; Mod Rheumatol 2008; 18:460-4 [30] | INF | Most early RA (1-5 years) | Active RA treated with INF/MTX (n=172).  INF discontinued after DAS28-ESR < 2.6 for at least 24 weeks (n=9) | DAS28-ESR < 2.6 for 24 weeks or longer | 9/9 maintained DAS28-ESR < 2.6 for at least 6 months after discontinuation; 7/9 for at least 12 months | Not reported |
| Soubrier, Rheumatology 2009; 48:1429-34 (GEUPARD) [31] | ADA | Early RA, MTX and biologic naïve | Active RA randomized (open label) to either MTX alone (n=32) or ADA/MTX (n=33).  If DAS28-ESR < 3.2 at Week 12, ADA was stopped. Patients followed to Week 52. | DAS28-ESR < 3.2 | 11/33 (33.3%) had DAS28-ESR < 3.2 off ADA through Week 52. | Not reported |
| Lagana, Int J Immunopathol Pharmacol 2009; 22:447-54 [32] | ETA | Early RA, poor prognostic factors, TNFi naïve | Active RA treated with ETA for 1 year (n=20).  ETA discontinued if DAS < 1.6 at 12 months (n=8) | DAS < 1.6 | 8 discontinued ETA. “Stable” at 24 months with all remaining in clinical remission. | Not applicable |
| Saleem, Ann Rheum Dis 2010; 69:1636-42 [33] | TNFi | Early RA (n= 27; < 2 years), or established RA (n= 20; 10 years) | Patients with DAS28 < 2.6 for 6 months or more while taking TNFi + MTX (range 6 -17 months), stopped TNFi and observed for 2 years. | DAS28 < 2.6 | Early patients 16/27 (59.2%) maintained remission.  Established patients 3/20 (15%) maintained remission.  Median time to flare 14 months and 4 weeks, respectively. | Not reported |
| Migliore, Int J Immunopathol Pharmacol 2010; 23:783-90 [34] | TNFi | Early RA | Patients with DAS28 < 3.2 on TNFi (duration 7 months; no duration of remission reported). Started on cyclosporin A at time of TNFi discontinuation and followed for 6 months. | DAS28 < 3.2 | 13/21 (61.9%) maintained DAS28 < 3.2.  8 (38%) had increase in DAS28 | Not reported |
| Migliore, Int J Immunopathol Pharmacol 2011; 24:167-74 [35] | TNFi | Early RA | Patients with DAS28 < 3.2 after 6-12 months of TNFi treatment, discontinued TNFi and started on cyclosporin A. Followed for 12 months.  No duration of low disease activity specified. | DAS28 < 3.2 | 21/50 (42%) with DAS28 < 3.2 at 12 months | Not reported |
| Harigai, Mod Rheumatol 2012; 22:814-22 (BRIGHT) [36] | ADA | Established RA (10.3 years) | Active RA, completed RCT, then open label extension with ADA for 26 weeks.  Those with DAS28-CRP < 2.7 at Week 26 were followed, either while continuing ADA (n=24) or after discontinuing ADA (n=22), for 52 wks.  34/46 (73.9%) not using DMARDs. One-half using glucocorticoids. | DAS28-CRP < 2.7 without biologics at Week 26 and 52 | 4/22 (18.1%) in discontinue group had DAS28-CRP < 2.7 at week 52. Others started biologics, relapsed or no data.  16/24 (66.6%) who continued ADA remained with DAS28-CRP < 2.7 at Week 26 and 52. | Not reported |
| Nam, Ann Rheum Dis 2014; 73:75-85 (IDEA) [37] | INF | Early RA (< 12 months), DMARD naïve | Active RA randomized to INF/MTX (n=55) or methylprednisolone/  MTX (n=57),  Patients on INF with DAS ≤ 1.6 for 6 months had INF stopped. | Change in Sharp score.  DAS ≤ 1.6 | 14 had prolonged remission on INF and stopped. 11/14 (78.6%) remained in remission off INF thru week 78 (duration not given, although most reported to be in the first year). Duration estimated to be 78-52=26weeks. | Not reported |
| Nam, Ann Rheum Dis 2014;73:1027-36 (EMPIRE) [38] | ETA | Early inflammatory arthritis, 1 or more tender or swollen joints, seropositive, DMARD naïve | Patients randomized to ETA/MTX (n=55) or placebo/MTX (n=55), followed for 52 weeks.  If no tender/swollen joints were present for > 26 weeks, ETA (or placebo) could be discontinued early. | No tender or swollen joints at Week 52 | 2 of 4 patients in ETA group who achieved sustained remission stopped ETA early (before Week 52).  Both (100%) had no tender/swollen joints at Week 78. | Not reported. |
| Tanaka Ann Rheum Dis 2015; 74:389-95  (HONOR) [39] | ADA | Established RA (mean 7.0; 8.6 years) | Patients treated with ADA/MTX and with DAS28-ESR < 2.6 for 6 months or longer without glucocorticoids and NSAIDs and stable MTX dose.  Patients and physicians elected to either continue ADA (n= 23) or discontinue ADA (n=52) and observe for 1 year. | DAS28-ESR < 2.6 at 1 year | Discontinue group 48% vs continue group 83% (p .006) with DAS28-ESR<2.6  Among those in deep remission (DAS28 < 1.98), 68% and 91% of discontinue and continue groups had DAS28 < 2.6 at 1 year. | Resuming ADA resulted in DAS28<3.2 in 90% by 6 months and 100% by 9 months (n=21) |
| Smolen, Ann Rheum Dis 2015; 74:843-50  (CERTAIN) [40] | CTZ | Established RA (4-5 years) | Patients with moderate/low disease activity on DMARDs, randomized to CTZ (n-96) or placebo (n=98) for 24 weeks.  Those with CDAI ≤ 2.8 at Week 20 and 24 stopped treatment (CTZ or placebo) and observed until Week 52 | CDAI ≤ 2.8 at week 20 and 24. | 14/17 (82.3%) who stopped CTZ lost remission at week 52 (28 weeks after discontinuation).  4/6 who stopped Placebo lost remission | 10/17 flared; all achieved remission or low disease activity with CTZ retreatment (10/10) |
| Tanaka, RMD Open 2016; 2:e000189  (HOPEFUL-2) [41] | ADA | Early (< 2 years), poor prognostic factors, MTX naïve | Active RA randomized to ADA/MTX or Placebo/MTX for 26 weeks, followed by open label ADA/MTX to week 52.  Patients could then elect to either continue open label ADA (n=106) or discontinue ADA (n=114), and followed for another 52 weeks.  Subset of pts with DAS28-ESR < 3.2 at week 46 and 52 examined separately: continue ADA group (n=73) vs discontinue ADA group (n=80) | DAS28-ESR < 3.2 | DAS28-ESR < 3.2 at week 104:  Continue ADA group: 71/73 (97.2%)  Discontinue ADA group: 64/80 (80.0%) | Not reported |
| Inui, Medicine 2018; 97(38):e12462  (RESUME) [42] | ETA | Established RA (6.1 years), biologic naïve | Patients with moderate or high activity started on ETA. Those who achieved DAS28-ESR < 3.2 within 2-6 months had ETA discontinued and were observed over 2 years (n=18). ETA restarted if low disease activity was lost. | DAS28-ESR < 3.2 | 5/18 (27.7%) remained off ETA with DAS28-ESR < 3.2 during follow-up (28%).  13/18 (72%) restarted ETA due to loss of low disease activity | 13/13 regained low disease activity after restarting ETA when assessed at 24 months |
| Tanaka, Ann Rheum Dis 2020; 79:94-102  (RRRR) [43] | INF | Established RA (> 3 years) | Active RA despite MTX, randomized to INF standard dose or to dose guided by serum TNF level for 54 weeks.    Those with SDAI ≤ 3.3 at week 54 had INF stopped in both groups (standard dose n=52; guided dose n=67) and followed for 1 year. | Off INF at 1 year. | 36/52 (standard dose) and 40/67 (guided dose) remained off INF (76/119; 63.8%) | Not reported |

*RA=rheumatoid arthritis; DAS28=Disease Activity Score 28; SDAI=Simplified Disease Activity Index; CDAI=Clinical Disease Activity Index; ESR= erythrocyte sedimentation rate; CRP=C-reactive protein; ADA=adalimumab; ETA=etanercept; INF=infliximab; CTZ=certolizumab; GOL=golimumab; TNFi=tumor necrosis factor inhibitor; RCT=randomized controlled trial; MTX=methotrexate; DMARD=disease-modifying anti-rheumatic drug; MRI=magnetic resonance imaging.

Supplemental table 4. Pooled proportions of patients having sustained remission/low disease activity after either

discontinuation or continuation of tumor necrosis factor inhibitor (TNFi) treatment in induction-withdrawal studies, stratified

by length of follow-up. CI = confidence interval.

|  |  | After TNFi Discontinuation | | |  | With TNFi Continuation | | |
| --- | --- | --- | --- | --- | --- | --- | --- | --- |
| Follow-up period (weeks) | Outcome | Number of studies | Pooled proportion (%) (95% CI) | I^2^ (%) (95% CI) |  | Number of studies | Pooled proportion (95% CI) | I^2^ (%) (95% CI) |
| **All studies** | | | | | | | | |
| 37-52 | DAS28<3.2 | 9 | 58 (45, 70) | 91.5 (81.2, 97.7) |  | 6 | 85 (70, 96) | 93.5 (82.9, 98.9) |
|  | DAS28<2.6 | 9 | 52 (35, 69) | 92.4 (82.0, 98.5) |  | 7 | 73 (61, 85) | 87.4 (68.1, 97.4) |
|  | SDAI≤3.3 | 4 | 40 (18, 64) | 94.8 (83.8, 99.6) |  | 4 | 62 (42, 80) | 90.8 (70.1, 99.3) |
|  |  |  |  |  |  |  |  |  |
| 24-36 | DAS28<3.2 | 2 | 36 (2, 82) | 94.1 (70.6, >99.9) |  | 1 | 44 (36, 52) | - |
|  | DAS28<2.6 | 4 | 73 (19, 100) | 93.2 (77.7, 99.5) |  | 1 | 34 (27, 42) | - |
|  | SDAI≤3.3 | 2 | 12 (8, 18) | 0 (0, 99.8) |  | 2 | 23 (16, 31) | 0 (0, 99.7) |
| **Studies with low or moderate risk of bias** | | | | | | | | |
| 37-52 | DAS28<3.2 | 5 | 59 (41, 75) | 92.7 (79.6, 99.1) |  | 5 | 81 (65, 94) | 93.1 (80.1, 99.2) |
|  | DAS28<2.6 | 7 | 57 (37, 76) | 93.8 (82.7, 99.0) |  | 5 | 72 (56, 86) | 91.2 (74.7, 98.9) |
|  | SDAI≤3.3 | 3 | 38 (11, 71) | 97.0 (88.8, 99.9) |  | 3 | 55 (35, 74) | 89.4 (58.9, 99.8) |
|  |  |  |  |  |  |  |  |  |
| 24-36 | DAS28<3.2 | 1 | 27 (9, 51) | - |  | 0 | - | - |
|  | DAS28<2.6 | 2 | 75 (44, 97) | 88.0 (39.5, 99.9) |  | 0 | - | - |
|  | SDAI≤3.3 | 2 | 12 (8, 18) | 0 (0, 99.8) |  | 2 | 23 (16, 31) | 0 (0, 99.7) |

Supplemental figure 1. Risk of bias evaluation of controlled trials among induction-withdrawal studies of tumor necrosis factor inhibitors using the Risk of Bias-2 tool.


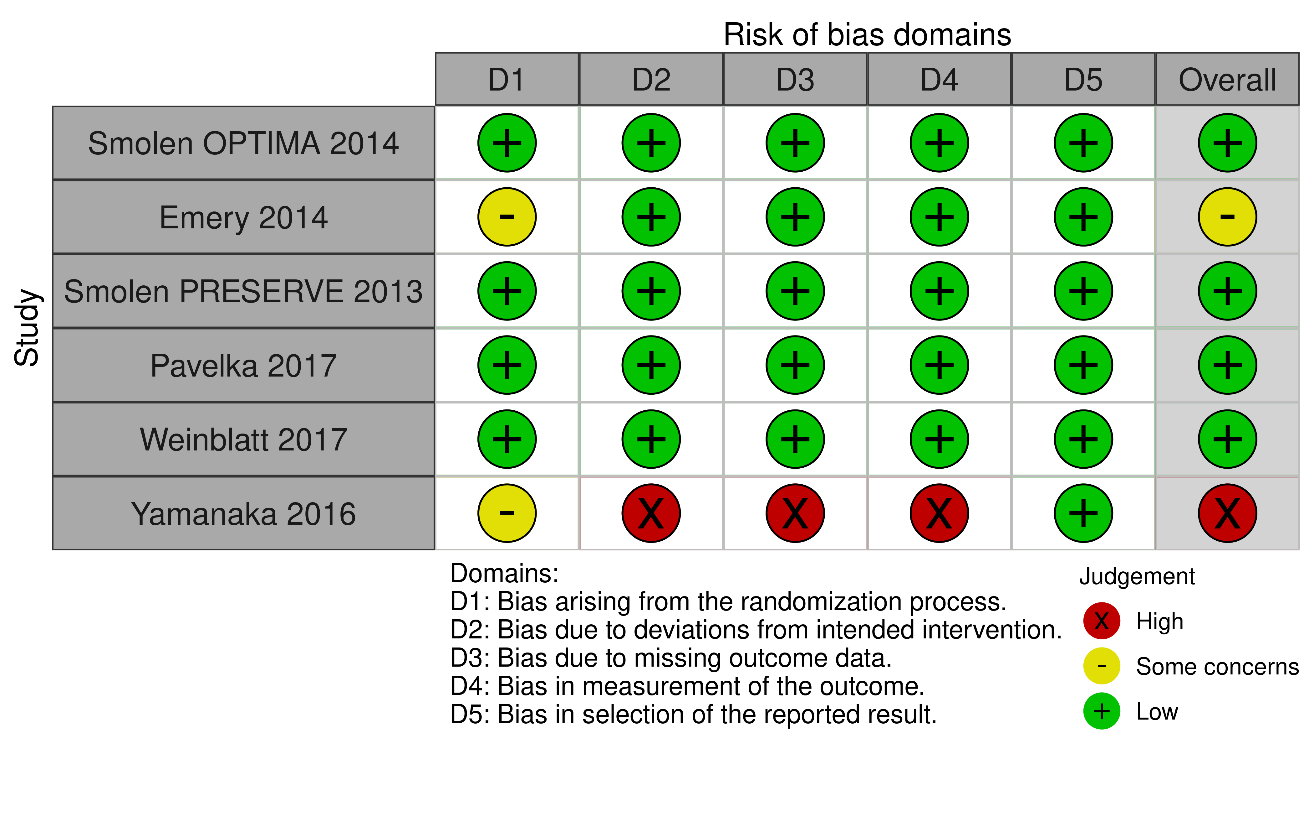


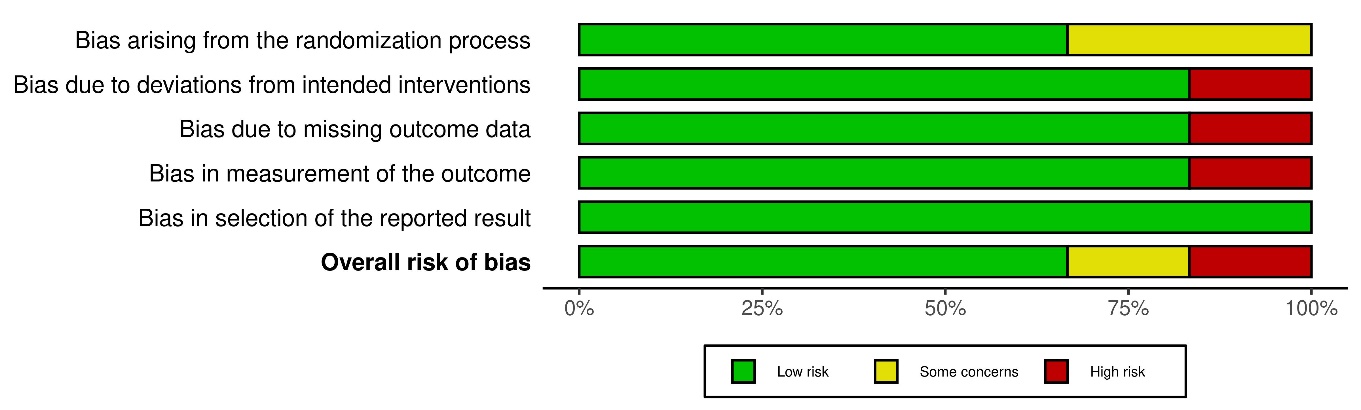


Supplemental figure 2. Risk of bias evaluation of observational studies of induction-withdrawal of tumor necrosis factor inhibitor treatment using the Risk of Bias in Non-randomised Studies of Interventions (ROBINS-I) tool.
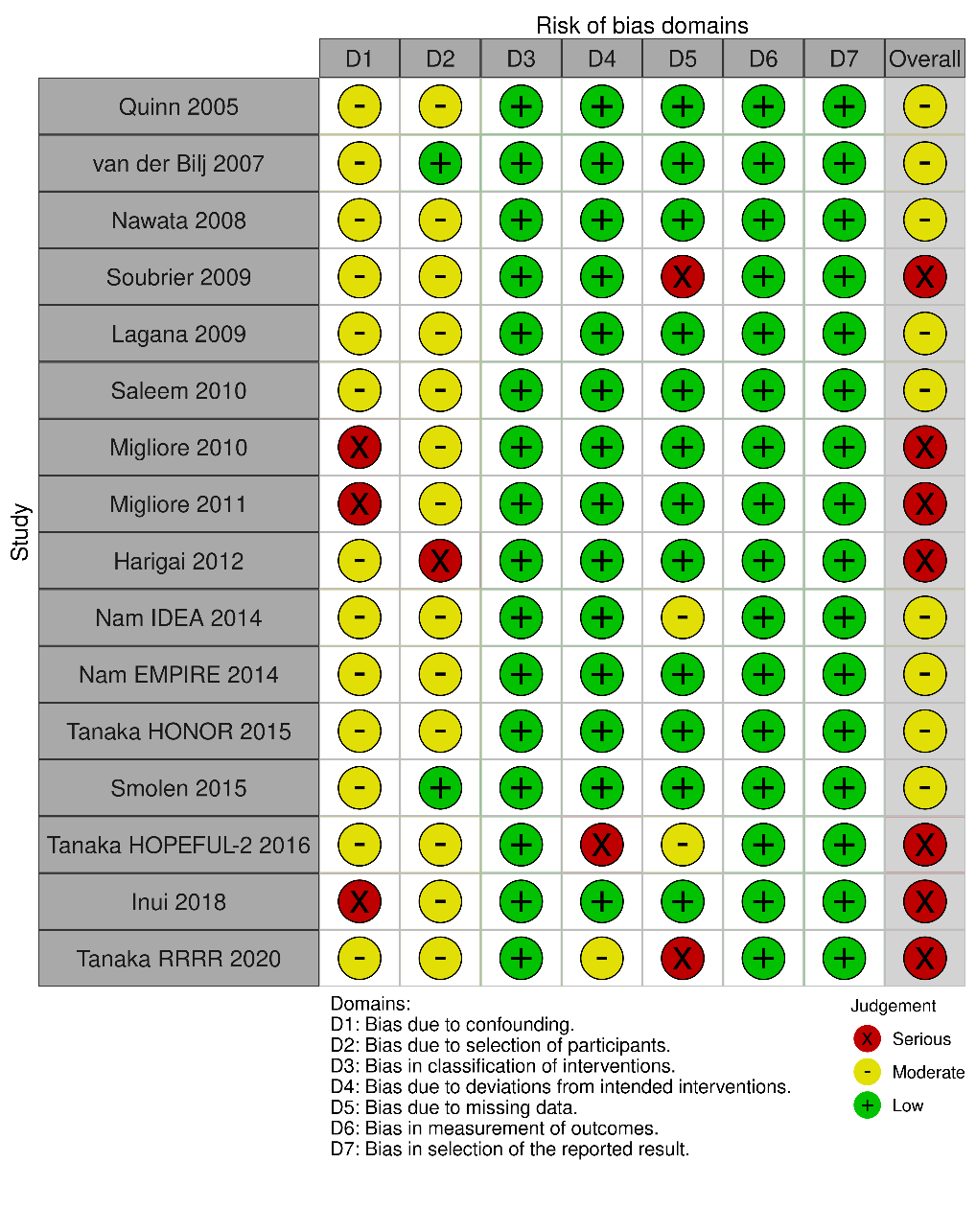


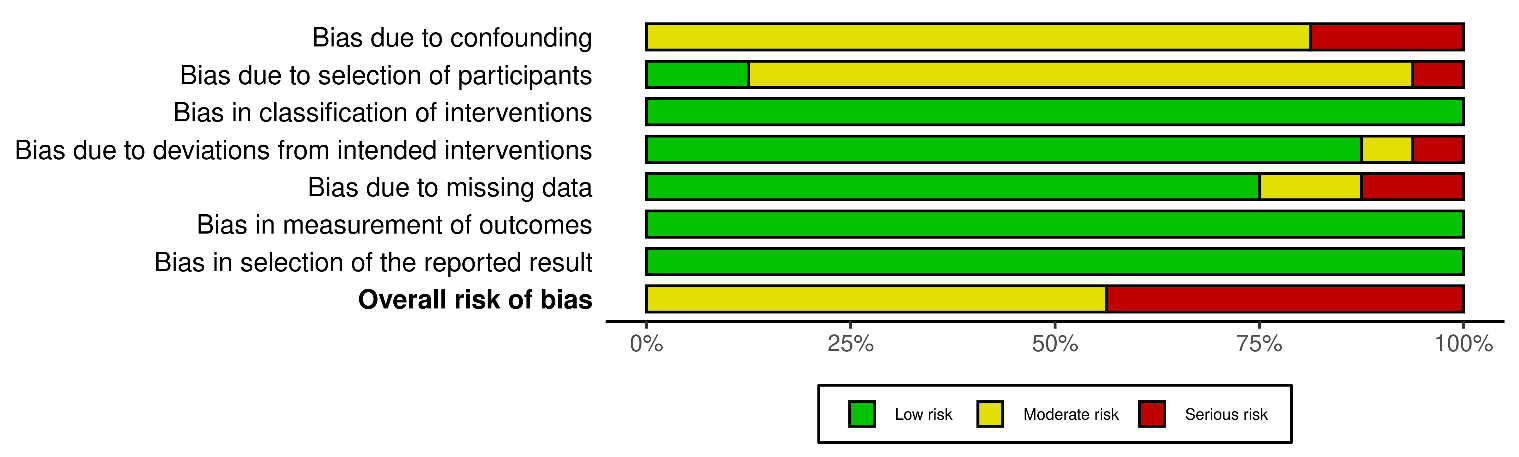


Supplemental table 5. Relative risks and risk differences of sustained remission/low disease activity with discontinuation

versus continuation of tumor necrosis factor inhibitor treatment in induction-withdrawal studies that reported both arms.

|  | Number of studies | Risk ratio (95% CI) | Risk difference (95% CI) |
| --- | --- | --- | --- |
| DAS28 < 3.2 |  |  |  |
| All studies | 7 | 0.69 (0.57, 0.83) | -22.2% (-31.4, -13.0) |
| Controlled trials | 5 | 0.65 (0.49, 0.87) | -21.9% (-34.2, -9.6) |
| Observational studies | 2 | 0.77 (0.63, 0.93) | -21.3% (-32.7, -9.8) |
|  |  |  |  |
| DAS28 < 2.6 |  |  |  |
| All studies | 8 | 0.58 (0.46, 0.71) | -27.3% (-35.0, -19.7) |
| Controlled trials | 6 | 0.60 (0.47, 0.75) | -24.9% (-33.0, -16.8) |
| Observational studies | 2 | 0.45 (0.22, 0.92) | -40.2% (-56.1, -24.4) |
|  |  |  |  |
| SDAI ≤ 3.3 |  |  |  |
| All studies | 6 | 0.59 (0.42, 0.82) | -18.4% (-26.5, -10.4) |
| Controlled trials | 4 | 0.53 (0.34, 0.82) | -19.6 (-29.4, -9.9) |
| Observational studies | 2 | 0.84 (0.59, 1.18) | -11.3 (-31.5, -8.9) |

Supplemental table 6. Detailed characteristics of studies of discontinuation of maintenance treatment with tumor necrosis

factor inhibitors.*

| Reference | Drug | Patients | Design | Remission criterion for discontinuation | Primary endpoint | Retreatment after relapse |
| --- | --- | --- | --- | --- | --- | --- |
| Van Vollenhoven, Ann Rheum Dis 2016; 75:52-8  (DOSERA) [44] | ETA | Established RA (13.6 years), taking ETA/MTX for at least 14 months, with DAS28-ESR ≤ 3.2 for at least 11 months. | Randomized  (double-blind) after run-in period to either continue ETA (n=23),  reduce ETA to 25mg/week (n=27), or discontinue ETA (i.e. placebo) (n=23)  59/73 (82%) with DAS28< 2.6 | DAS28-ESR ≤ 3.2 for ≥ 11 months | Nonfailure at Week 48.  Failure = DAS28-ESR > 5.1, or DAS28-ESR > 3.2 with increase by 1.2 or more, or increase by 0.6 or more twice, or MD or patient decision  Continue group 52%; Reduced dose group 44%;  Discontinue group 13% | 19/20 (95%) in discontinue group regained DAS28-ESR < 3.2 with retreatment by 20 weeks. |
| Emery, Ann Rheum Dis 2020; 79:1023-30  (PREDICTRA) [45] | ADA | Established RA (12.9 y) on ADA ≥ 12 months with DAS28 < 2.6 for at least 6 months. | Randomized (double- blind) after run-in period to ADA 40 q 3 weeks (n=102) or placebo (n=20). Followed for 36 weeks. | DAS28-ESR < 2.6 for ≥ 6 months | Flare: DAS28-ESR increase by 1.2, or DAS28-ESR ≥ 2.6 and increase by 0.6  Flare:  ADA group 36%  Discontinue group 45%  DAS28-ESR < 2.6 at week 40:  86% ADA vs 85% Placebo among those still enrolled | 4/8 pts in discontinue group regained remission by 16 weeks after restarting ADA |
| Curtis, Arthritis Rheum 2021; 73:759-68  (SEAM-RA) [46] | ETA | Established RA (9-11 years), with SDAI ≤ 3.3 while taking ETA/MTX at screening | Open label run-in with ETA/MTX for 24 weeks (1 visit with SDAI > 3.3 allowed), then randomized (double-blind) to Placebo/MTX (n=101), Placebo/ETA (n=101), or ETA/MTX (n=51), followed for 48 weeks | SDAI ≤ 3.3 for 24 weeks | SDAI ≤ 3.3 at 48 weeks: ETA alone 49.5%;  ETA/MTX 52.9%;  MTX alone (discontinue group) 28.7%  Probability of no worsening by week 48:  38% discontinue group,  59.6% ETA group,  65.2% ETA/MTX group | Of 52 patients in the Discontinue group who were retreated, 71% of regained SDAI ≤ 3.3 remission by study end (44 weeks); 96% regained low disease activity. |
| Chatzidionysiou, RMD Open 2016; 2:e000133  (ADMIRE) [47] | ADA | Established RA (7.6 and 10 years), seropositive or erosive, on ADA/MTX for at least 6 months, and DAS28<2.6 at least 3 months | Randomized (open label) to continue ADA/MTX (n =17), or stop ADA and continue MTX alone (n= 16), followed for 52 weeks. | DAS28 < 2.6 ≥ 3 months | DAS28<2.6 at Week 28:  Continue group 94%  Discontinue group 33%  (p = .001)  50% of continue group and 73% of discontinue group had DAS28> 2.6 at some time before week 28 | Restart of ADA resulted in remission in 8/9 patients within 12 weeks. |
| Ghiti Moghadam, Arthritis Rheum 2016; 68:1810-7  (POET) [48] | Any TNF (90% either ADA or ETA) | Established RA (12.0 and 11.1 y), on TNFi for 12 months or more, DAS28-ESR<3.2 for 6 months or more, or rheumatologist judgment of low activity. | Randomized (open label) to continue TNFi (n=286) or discontinue TNFi (n= 531).  653 (79.9%) with DAS28-ESR < 2.6 at baseline.  Followed for 12 months. | DAS28-ESR < 3.2 ≥ 6 months | Flare: DAS28-ESR ≥3.2 and increase by > 0.6 from baseline at 12 months:  Continue group 18.2%  Discontinue group 51.2%  Among those in DAS28<2.6 at baseline, 56.9% (128/255) of continue group and 29.7% (127/428) of discontinue group remained in DAS28 remission | 252 restarted TNFi after flare with discontinuation.  195 did so within 26 weeks and had adequate follow-up.  132/195 (67%) achieved DAS28-ESR< 2.6 and  165/195 (85%) achieved DAS28-ESR<3.2 by week 52 |
| Brocq, Joint Bone Spine 2009; 76:350-5 [49] | TNFi | Established RA (11.3 y), on TNFi treatment with DAS28 < 2.6 for at least 6 months, with no more than prednisone 5 mg/d and without NSAIDs | Open label discontinuation (n=21)  1 died soon after; data on 20 patients.  6 were not on DMARDs. | DAS28 < 2.6 for ≥ 6 months | Relapse DAS28 > 3.2:  11/20 (55%) relapsed within 6 months;  15/20 (75%) relapsed within 12 months. | 15/15 regained remission with resumption of TNFi by 20 weeks |
| Tanaka, Ann Rheum Dis 2010; 69:1286-91 (RRR) [50] | INF | Established RA (5.9 y), treated with INF, and DAS28-ESR < 3.2 for at least 24 weeks. | Open label discontinuation (n=114), followed for 52 weeks. | DAS28-ESR < 3.2 for at least 24 weeks | 56/102 (54.9%) with DAS28_ESR < 3.2 off INF at 1 year  44/102 (43%) with DAS28-ESR <2.6 | 32 patients retreated after relapse, and “majority” regained DAS28-ESR<3.2 by 24 weeks (data only in figure). |
| Saleem, Ann Rheum Dis 2010; 69:1636-42 [33] | TNFi | Early RA (n= 27; < 2 years), and Established RA (n= 20; 10 years) with DAS28 < 2.6 for 6 months or more while taking TNFi + MTX (range 6 -17 mo). | Open label discontinuation of TNFi and observed for 2 years. | DAS28 < 2.6 | Early RA patients: 16/27 (59%) maintained remission.  Established RA patients: 3/20 (15%) maintained remission.  Median time to flare 14 months and 4 weeks, respectively. | Not reported |
| Iwamoto, Arthritis Care Res 2014; 66:1576-81 [51] | Biologics (32/42 TNFi; 10/42 Tocilizumab) | Established RA (8.2 y) with DAS28 < 2.6 for 3 months or longer | Patients discontinued biologic and followed for 6 months | DAS28 < 2.6 | Relapse: DAS28 > 3.2 and treatment escalated:  16/42 (38%) relapsed (12/32 on TNFi; 37.5%).  19/32 (59%) on TNFi remained with DAS28 < 2.6 at 6 months | Not reported |
| Kurasawa, Mod Rheumatol 2014; 24:561-6 [52] | INF | Established RA (7.1 y), with DAS28-CRP < 2.6 for at least 6 months on INF/MTX. | All discontinued INF.  Randomized to add bucillamine (n=24) or continue MTX alone (n= 31)  Followed for 2 years. | DAS28-CRP < 2.6 for at least 6 months | Flare = DAS28-CRP ≥ 2.6 and DAS28-ESR ≥3.2:  In MTX group at 12 months 55% without flare; at 24 months 63% flared.  45% with loss of remission at 12 months. | Not reported |
| Kadar, Clin Rheumatol 2014; 33:329-33 [53] | TNFi | Established RA, treated with TNFi | Discontinuation specifically due to long-term remission (n=6) | Remission (DAS-28<2.6) | 6/6 remained in remission during follow-up (range 3-55 months)  6/6 for at least 3 months;  5/5 for at least 6 months. | Not reported |
| Kavanaugh, Ann Rheum Dis 2015; 74:1150-5 [54] | TNFi | CORRONA registry. Established RA (8 years), taking first TNFi, CDAI ≤ 10 at time of discontinuation (no duration specified) | Patients who stopped TNFi and had no other treatment added (n=717). Followed with questionnaire and clinic visits.  300 (41.8%) on TNFi without a DMARD | CDAI ≤ 10 | Failure=CDAI > 10 or any medication increase or addition:  At 12 months, 73.4% remained without failure;  At 24 months, 42.2% remained without failure. | Not reported |
| Yoshida, Rheumatology 2016; 55:286-90 [55] | Any biologic (82% TNFi) | NINJA registry. Established RA, treated with biologic and CDAI ≤ 2.8 on at least occasion before discontinuation. | Patients who stopped treatment, followed for 1 to 4 years) (n=46; 38 on TNFi) | CDAI ≤ 2.8 on at least occasion | Failure = CDAI > 2.8, resume treatment with biologic, or other treatment escalation: 67% reached failure over 1 year | Not reported |
| Kawashiri, Arthritis Res Ther 2017; 19:108 [56] | TNFi  ETA 10%  ADA 23%  INF 47%  GOL 13%  CTZ 7% | Established RA, treated with TNFi for 6 months or longer, with DAS28-ESR < 3.2 for at least 3 months, not taking glucocorticoids, Doppler ultrasound not >1 in any single joint | Open label discontinuation (n=30) | DAS28-ESR < 3.2 for at least 3 months | 14/30 (46%) without treatment escalation at 12 months | Not reported |
| Kimura, Inflamm Regen 2019; 39:5 [57] | ADA | Established RA, with DAS28-ESR < 2.6 for at least 6 months while treated with ADA | Of 39 patients with sustained remission,  4/39 discontinued ADA. | DA28-ESR < 2.6 | 3/4 patients lost remission (‘flare”) at 3-8 months after discontinuation.  1 patient in remission for 29 months.  7/29 patients (24%) who continued ADA also flared during follow-up. | 2/3 patients regained remission within 1 month of restarting ADA. The remaining patient did not regain remission. |
| Ito, Intern Med 2019; 58:511-9 [58] | ADA | Established RA, clinical remission for > 2 years on ADA or patient request if sooner  Mean DAS28 at baseline 3.4. | Of 26 patients who discontinued ADA, data reported on 20 patients followed at least 6 months | Clinical judgment | DAS28-CRP < 2.7 at 6 months in 19/20 (95%)  DAS28-CRP < 2.3 at 6 months in 18/20 (90%) | 1/1 regained remission after restarting ADA |
| Naniwa, Mod Rheum 2020; 30:948-58 [59] | TNFi | Early RA, sustained low activity on treatment with TNFi/MTX for 6 months or longer, with SDAI ≤ 11. | Open label discontinuation of TNFi, followed for 12 months (n=95). | SDAI ≤ 11 | Flare = DAS28 increase ≥ 1.2 on 2 consecutive visits and treatment escalation (DMARD or glucocorticoids):  33/95 patients (34.7%) with flare in first year  (65% without flare over follow-up) | 23/26 patients (88%) who restarted TNFi after flare regained disease control by 12 months. |
| Takai, Intern Med 2020; 59:1963-70 [60] | INF | Established RA (8.4 y), in clinical remission for 2 years (or shorter if discontinued due to financial reasons) while treated with INF | Patients discontinued INF (n=18) and follow-up for 96 weeks. Mean INF treatment for 45.8 months | DAS28-ESR < 2.6, usually > 2 years | 55.6% with DAS28-ESR < 2.6 while off INF at 48 weeks; 44.4% at 96 weeks  12/18 had csDMARDs increased at time of INF discontinuation. | Of 10 patients who flared, 4 treated with DMARD escalation, 6 restarted TNFi. Follow-up reported on 5/6, with 5/5 regaining clinical remission within 5 weeks. |
| Kameda, Sci Report 2021;11:6865 [61] | TNFi | Established RA (median 5.2 years), SDAI ≤ 3.3 for at least 3 months while on biologic treatment. | Open label discontinuation of biologic, followed for 2 years.  (N=26 on TNFi) | SDAI ≤ 3.3 | Relapse = DAS28-ESR ≥ 3.2 and increase by 0.6 or more from baseline: Relapse in 14/26 (53.8%), median 115 days, all within first year of discontinuation. | Not reported |
| Ochiai, Mod Rheumatol 2021; 31:790-5 [62] | TNFi | Registry study of established RA patients, treated with biologics and DAS28-ESR < 3.2 at discontinuation (duration of low activity not specified) | Patients who discontinued treatment, followed for 12 months (n=39). | DAS28-ESR < 3.2 (no duration reported) | 22/39 (56%) off TNFi at 12 months | 8 patients restarted TNFi after relapse. Follow-up at 1 year after restart available for 7, with 7/7 having DAS28-ESR<2.6. |
| Burkard, Rheumatol Adv Pract 2021; 13:5:rkab087 [63] | Biologic or targeted DMARD  (70% TNFi) | SCQM Registry study, Established RA patients treated with biologic or targeted DMARD with treatment stop attributed to remission (no duration specified). DAS28 < 2.6 or RADAI < 1.5 within 4 months prior to stop. | Patients who discontinued treatment, followed for 1 year (n=302 episodes; 212 with TNFi) | DAS28 < 2.6 or RADAI < 1.5 | 148/212 (70%) who stopped TNFi restarted biologic over a median of 2 years | Not reported |
| Nagatani Sci Rep 2021;11:20771 [64] | TNFi | Established RA, treated with TNFi and with DAS28-CRP < 2.3 for at least 12 months. | Open label discontinuation, other treatments unchanged, followed for 24 months (n=34) | DAS28-CRP < 2.3 for at least 12 months. | DAS28-CRP ≥ 2.3 during follow-up:  21/34 (62%) relapsed, 13/34 (38%) remained in remission for 24 mo.  Almost all relapses occurred by 12 months. | Not reported |

*RA=rheumatoid arthritis; DAS28=Disease Activity Score 28; SDAI=Simplified Disease Activity Index; CDAI=Clinical Disease Activity Index; ESR= erythrocyte sedimentation rate; CRP=C-reactive protein; ADA=adalimumab; ETA=etanercept; INF=infliximab; CTZ=certolizumab; GOL=golimumab; TNFi=tumor necrosis factor inhibitor; RCT=randomized controlled trial; MTX=methotrexate; DMARD=disease-modifying anti-rheumatic drug.

Supplemental table 7. Pooled proportions of patients having sustained remission/low disease activity after either

discontinuation or continuation of maintenance treatment with tumor necrosis factor inhibitor (TNFi), stratified by length of

follow-up.

|  |  | After TNFi Discontinuation | | |  | With TNFi Continuation | | |
| --- | --- | --- | --- | --- | --- | --- | --- | --- |
| Follow-up period (weeks) | Outcome | Number of studies | Pooled proportion (%) (95% CI) | I^2^ (%) (95% CI) |  | Number of studies | Pooled proportion (95% CI) | I^2^ (%) (95% CI) |
| **All studies** | | | | | | | | |
| 37-52 | DAS28<3.2 | 10 | 48 (38, 59) | 89.3 (73.1, 97.1) |  | 2 | 69 (38, 94) | 88.9 (44.3, 99.9) |
|  | DAS28<2.6 | 6 | 47 (33, 62) | 86.0 (60.0, 97.6) |  | 2 | 64 (46, 82) | 74.6 (0, 99.9) |
|  | SDAI≤3.3 | 2 | 46 (14, 80) | 96.1 (80.3, 99.9) |  | 1 | 53 (39, 67) | - |
|  |  |  |  |  |  |  |  |  |
| 24-36 | DAS28<3.2 | 2 | 85 (30, 100) | 94.1 (70.7, 100) |  | 0 | - | - |
|  | DAS28<2.6 | 4 | 75 (39, 99) | 85.4 (53.0, 99.0) |  | 2 | 78 (43, 99) | 86.4 (31.9, 99.9) |
|  | SDAI≤3.3 | 0 | - | - |  | 0 | - | - |
| **Studies with low or moderate risk of bias** | | | | | | | | |
| 37-52 | DAS28<3.2 | 4 | 39 (24, 57) | 66.8 (0, 97.6) |  | 1 | 52 (31, 73) | - |
|  | DAS28<2.6 | 4 | 53 (38, 68) | 70.4 (12.9, 98.5) |  | 1 | 75 (58, 90) | - |
|  | SDAI≤3.3 | 2 | 46 (14, 80) | 96.1 (80.3, 99.9) |  | 1 | 53 (39, 67) | - |
|  |  |  |  |  |  |  |  |  |
| 24-36 | DAS28<3.2 | 1 | 59 (41, 76) | - |  | 0 | - | - |
|  | DAS28<2.6 | 1 | 60 (37, 81) | - |  | 1 | 62 (53, 72) | - |
|  | SDAI≤3.3 | 0 | - | - |  | 0 | - | - |

Supplemental figure 3. Risk of bias (ROB2) evaluation of controlled trials of discontinuation of maintenance treatment with tumor necrosis factor inhibitors.


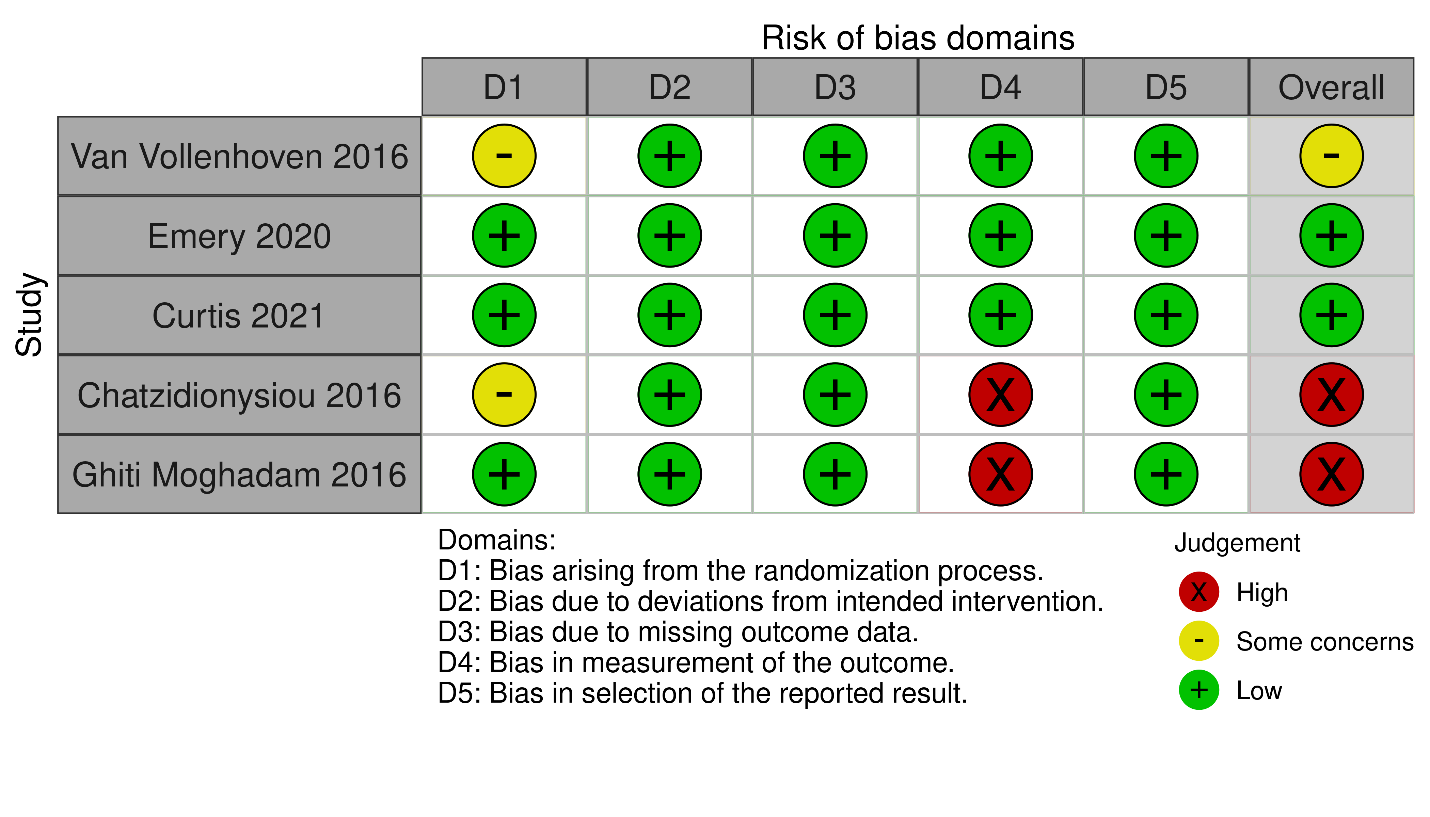


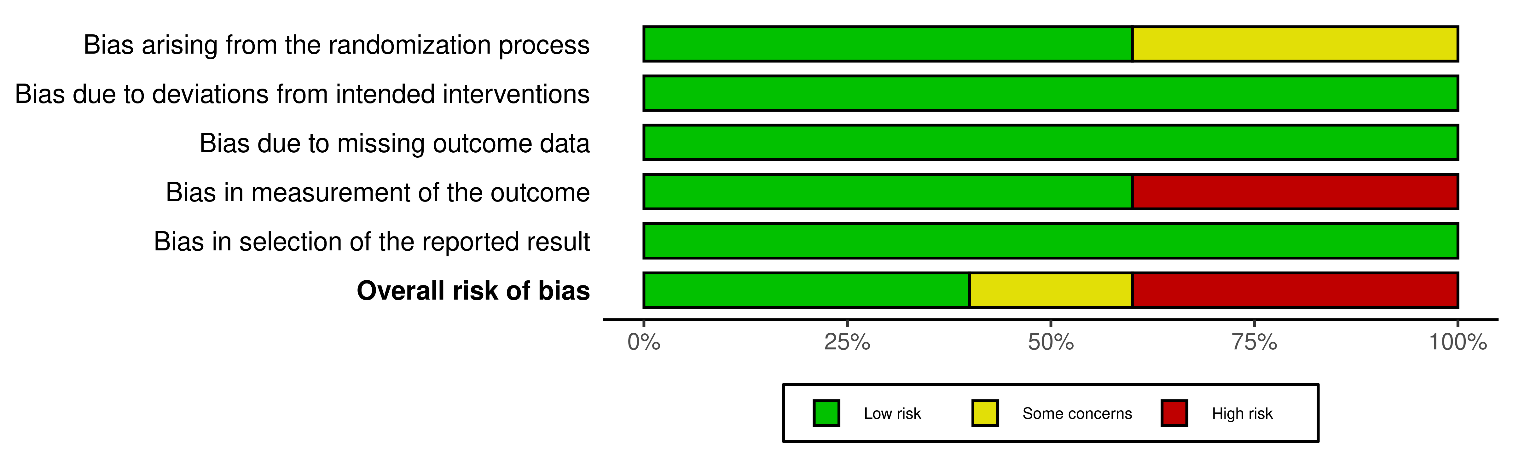


Supplemental figure 4. Risk of bias evaluation in observational studies of discontinuation of maintenance treatment with tumor necrosis factor inhibitors, using the Risk of Bias In Non-randomised Studies of Interventions (ROBINS-1) tool.


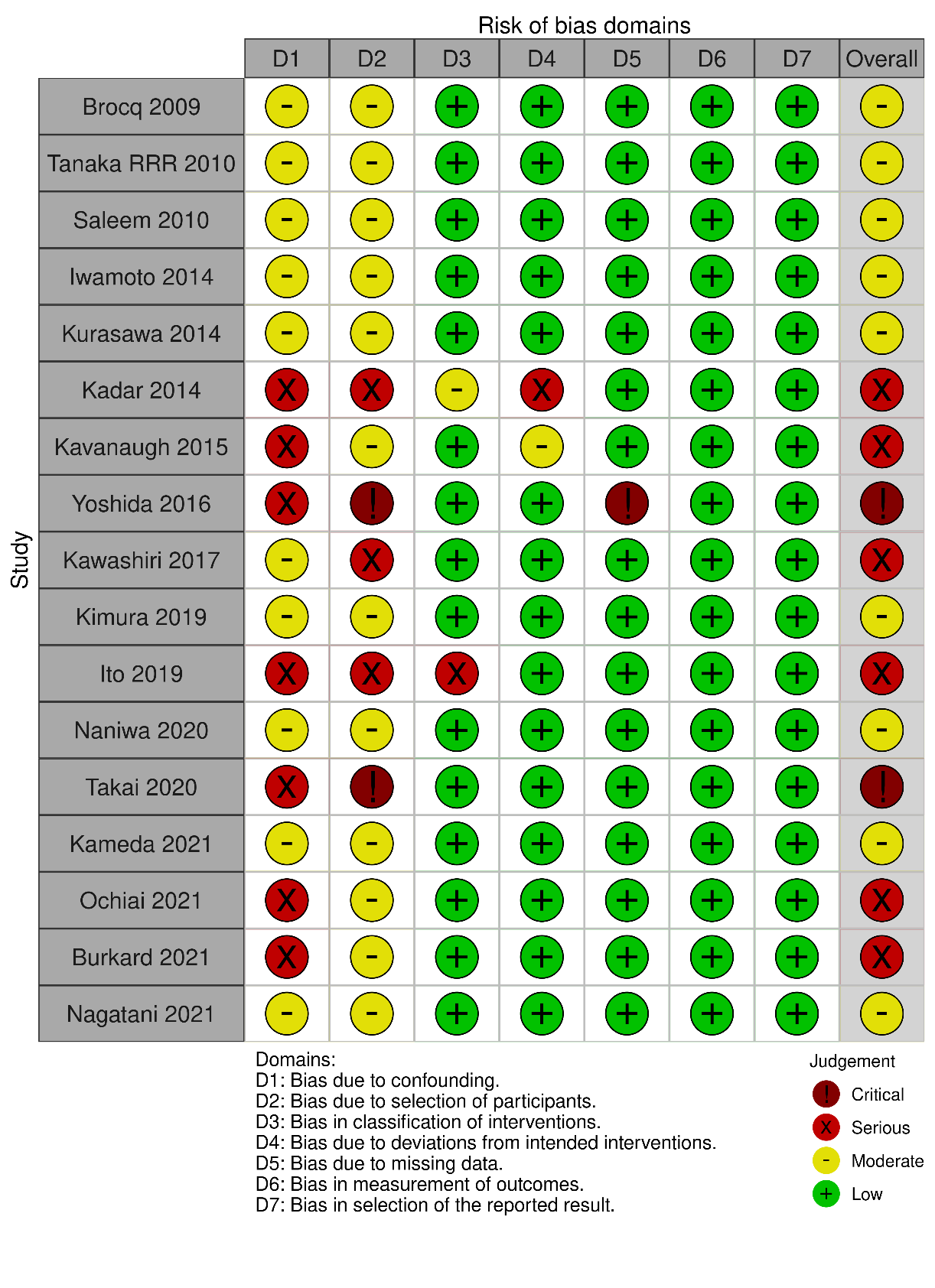


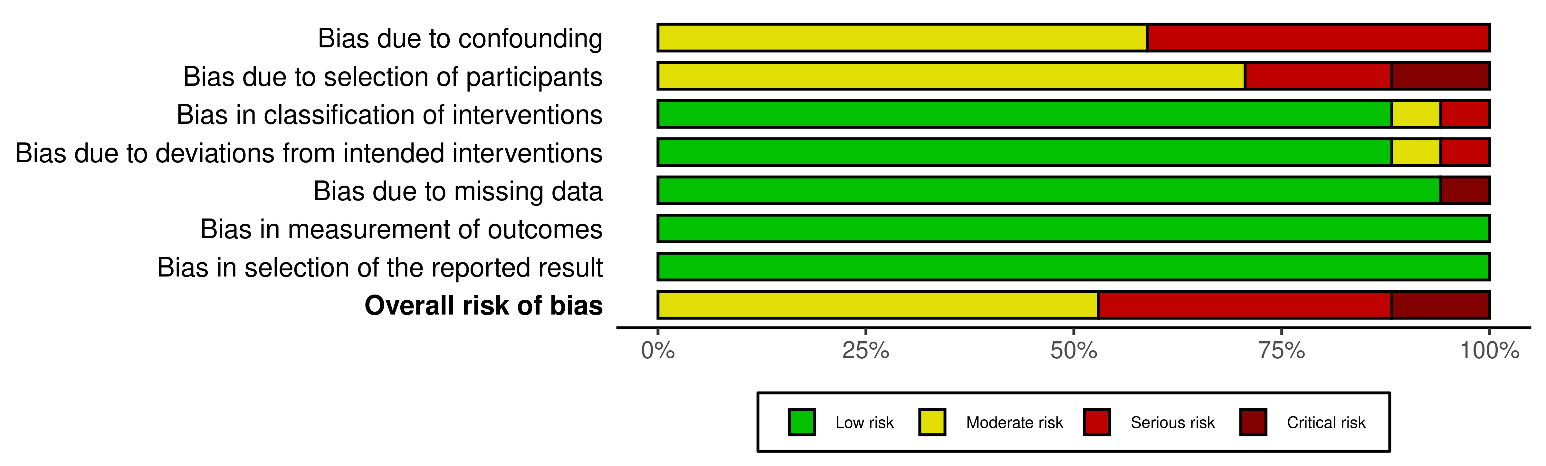


Supplemental table 8. Relative risks and risk differences of sustained remission or low disease activity with

discontinuation versus continuation of tumor necrosis factor inhibitor treatment in maintenance discontinuation studies that

reported both arms.

|  | Number of studies | Risk ratio (95% CI) | Risk difference (95% CI) |
| --- | --- | --- | --- |
| DAS28 < 3.2 |  |  |  |
| All discontinuation studies | 2 | 0.47 (0.21, 1.01) | -33.4% (-39.4, -27.4) |
| Controlled trials | 2 | 0.47 (0.21, 1.01) | -33.4% (-39.4, -27.4) |
| Observational studies | 0 | - | - |
|  |  |  |  |
| DAS28 < 2.6 |  |  |  |
| All studies | 4 | 0.57 (0.36, 0.90) | -32.1% (-53.6, -10.6) |
| Controlled trials | 3 | 0.59 (0.36, 0.96) | -29.1% (-53.6, -4.7) |
| Observational studies | 1 | 0.33 (0.06, 1.83) | -50.9% (-96.1, -5.7) |
|  |  |  |  |
| SDAI ≤ 3.3 |  |  |  |
| All studies | 1 | 0.53 (0.35, 0.78) | -26.2% (-42.4, -9.9) |
| Controlled trials | 1 | 0.53 (0.35, 0.78) | -26.2% (-42.4, -9.9) |
| Observational studies | 0 | - | - |

Supplemental table 9. Predictors of successful discontinuation by study.

| Predictor | Induction-Withdrawal Studies | | | | Maintenance Treatment Discontinuation Studies | | | |
| --- | --- | --- | --- | --- | --- | --- | --- | --- |
|  | Reference | Weight | Association with Successful Discontinuation | Standardized mean difference, Odds Ratio, or Hazard Ratio | Reference | Weight | Association with Successful Discontinuation | Standardized mean difference, Odds Ratio, or Hazard Ratio |
| **Age, continuous** | Tanaka 2016 [41] HOPEFUL-2 | 0.296 | Success 54.1 yrs  Failure 56.3 yrs | -0.18 (-0.72, 0.36) | Chatzidionysiou  2016 [47] | 0.02 | Success median 65 yrs  Failure median 63.5 yrs | -0.89 (-2.19, 0.41) |
|  | Tanaka 2015 [39] HONOR | 0.296 | Success 57.1 yrs  Failure 62.6 yrs | -0.48 (-1.03, 0.06) | Tanaka 2010 [50] RRR | 0.22 | Success 49.5 yrs  Failure 56.1 yrs | -0.52 (-0.92, -0.13) |
|  | Harigai 2012 [36] | 0.188 | Success 58.3 yrs  Failures 54.9 yrs | 0.24 (-0.86, 1.34) | Brocq 2009 [49] | 0.034 | Success 61.4 yrs  Failure 59.7 yrs | 0.12 (-0.89, 1.13) |
|  | Migliore 2010 [34] | 0.218 | Success 55.4 yrs  Failure 44.5 yrs | 1.28 (0.34, 2.22) | Iwamoto 2014 [51] | 0.086 | Success 58.0 yrs  Failure 62.3 yrs | -0.32 (-0.96, 0.31) |
|  |  |  |  |  | Takai 2020 [60] | 0.04 | Success median 59.8 yrs  Failure median 63.3 yrs | -0.04 (-0.96, 0.89) |
|  |  |  |  |  | Kurasawa 2014 [52] | 0.056 | Success 50.6 yrs  Failure 54.0 yrs | -0.25 (-1.03, 0.52) |
|  |  |  |  |  | Kameda 2021 [61] | 0.074 | Success median 69 yrs  Failure median 69 yrs | 0.06 (-0.61, 0.74) |
|  |  |  |  |  | Naniwa 2020 [59] | 0.19 | Success median 57 yrs  Failure median 56 yrs | 0.17 (-0.24, 0.60) |
|  |  |  |  |  | Nagatani 2021 [64] | 0.08 | Success median 60 yrs  Failure median 59 yrs | -0.14 (-0.79, 0.50) |
|  |  |  |  |  | Ochiai 2021 [62] | 0.095 | Success median 52 yrs  Failure median 46 yrs | 0.04 (-0.56, 0.64) |
|  |  |  |  |  | Kawashiri 2017 [56] | 0.09 | Success median 53 yrs  Failure median 55 yrs | -0.16 (-0.78, 0.46) |
| **Summary SMD** |  |  |  | **0.12 (-0.56, 0.82) p=0.72** |  |  |  | **-0.15 (-0.34, 0.03)**  **p=0.10** |
| **I^2^** |  |  |  | **71.6** |  |  |  | **0** |
|  |  |  |  |  |  |  |  |  |
| **Age categorical, older vs younger** | Emery 2018 [65] PRIZE | 0.457 | Younger age associated with successful discontinuation; OR estimated from figure | 0.50 (0.26, 0.95) | Ghiti Moghadam 2019 [69] | 1.0 | No association with age ≤ 60 | 0.85 (0.57, 1.25) |
|  | Tanaka 2020 [43] RRRR | 0.542 | Combined arms. Age ≥ 65 vs < 65 | 0.65 (0.36, 1.17) |  |  |  |  |
|  | Migliore 2011 [35] |  | Unable to calculate | No data |  |  |  |  |
| **Summary OR** |  |  |  | **0.58 (0.37, 0.89) p=0.01** |  |  |  | **0.85 (0.57, 1.25)**  **p=0.83** |
| **I^2^** |  |  |  | **0** |  |  |  | **-** |
|  |  |  |  |  |  |  |  |  |
| **Age categorical, older vs younger, HR** | Van den Broek 2011 [66] BeST | 1.0 | HR inverted | 1.00 (0.98, 1.02) | Kavanaugh 2015 [54] | 0.684 | HR inverted. Age ≥ 60 vs < 60, less likely to fail. | 1.02 (0.86, 1.19) |
|  |  |  |  |  | Yoshida 2016 [55] | 0.315 | HR inverted. older less likely to fail. | 1.06 (0.84, 1.33) |
| **Summary HR** |  |  |  | **1.00 (0.98, 1.02) p=0.99** |  |  |  | **1.03 (0.91, 1.17)**  **P=0.62** |
| **I^2^** |  |  |  | **-** |  |  |  | **0** |
|  |  |  |  |  |  |  |  |  |
| **Women vs Men** | Emery 2018 [65] PRIZE | 0.321 | Only OR provided, values estimated from figure | OR 0.7 (0.32, 1.5) | Ghiti Moghadam 2019 [69] | 0.609 | Only OR provided | 1.07 (0.71, 1.60) |
|  | Tanaka 2016 {41] HOPEFUL-2 | 0.091 | Success 84% women  Failure 81% women | 1.24 (0.30, 5.18) | Tanaka 2010 [50] RRR | 0.104 | Success 75% women  Failure 82.6% women | 0.63 (.23, 1.67) |
|  | Tanaka 2020 {43] RRRR | 0.539 | Combined arms | 0.63 (0.35, 1.14) | Brocq 2009 [49] | 0.016 | Success 86% women Failure 69% women (error in numbers) | 2.0 (0.17, 22.94) |
|  | Harigai 2012 [36] | 0.03 | Success 75% women  Failure 60% women | 2.33 (0.19, 27.56) | Iwamoto 2014 [51] | 0.032 | Success 75% women  Failure 87.5% women | 0.43 (0.07, 2.45) |
|  | Migliore 2010 [34] | 0.017 | Success 100% women  Failure 90% women | 3.63 (0.13, 99.84) | Takai 2020 [60] | 0.01 | Success 75% women  Failure 100% women | 0.12 (0.005, 3.00) |
|  |  |  |  |  | Kurasawa 2014 [52] | 0.021 | Success 80% women  Failure 88.2% women | 0.53 (0.06, 4.53) |
|  |  |  |  |  | Kameda 2021 [61] | 0.028 | Success 85.7% women  Failure 80% women | 1.50 (0.23, 9.58) |
|  |  |  |  |  | Naniwa 2020 [59] | 0.067 | Success79% women  Failures 87.8% women | 0.52 (0.15, 1.74) |
|  |  |  |  |  | Nagatani 2021 [64] | 0.04 | Success 71.4% women  Failure 84.6% women | 0.45 (0.09, 2.19) |
|  |  |  |  |  | Ochiai 2021 [62] | 0.04 | Success 80% women  Failure 83.3% women | 0.80 (0.16, 3.88) |
|  |  |  |  |  | Kawashiri 2017 [56] | 0.031 | Success 90.4%  Failure 73.6% | 3.39 (0.57, 20.10) |
|  |  |  |  |  | Lurati 2016 [70] | 0 | Women less likely to have successful discontinuation. No CI reported | 0.76 |
| **Summary OR** |  |  |  | **0.75 (0.49, 1.15)**  **p=0.19** |  |  |  | **0.91 (0.67, 1.25)**  **p=0.57** |
| **I^2^** |  |  |  | **0** |  |  |  | **0** |
|  |  |  |  |  |  |  |  |  |
| **Women** | Van den Broek 2011 [66] BeST | 1.0 | Inverted HR. | 0.91 (0.50, 1.66) | Kavanaugh 2015 [54] | 0.96 | Inverted HR | 0.97 (0.80, 1.17) |
|  |  |  |  |  | Yoshida 2016 [55] | 0.04 | Inverted HR | 1.31 (0.49, 3.44) |
| **Summary HR** |  |  |  | **0.91 (0.50, 1.66)**  **p=0.97** |  |  |  | **0.98 (0.82, 1.19) p=0.84** |
| **I^2^** |  |  |  | **-** |  |  |  | **0** |
|  |  |  |  |  |  |  |  |  |
|  |  |  |  |  |  |  |  |  |
| **Duration of RA, continuous** | Tanaka 2016 {41]  HOPEFUL-2 | 0.32 | Success 1.2 yrs  Failure 1.3 yrs | -0.30 (-0.85, 0.24) | Chatzidionysiou  2016 [47] | 0.021 | Success median 5.5 yrs  Failure median 10.9 yrs | -0.39 (-1.67, 0.87) |
|  | Tanaka 2015 [39] HONOR | 0.32 | Success 6.6 yrs  Failure 9.8 yrs | -0.31 (-0.86, 0.22) | Tanaka 2010 [50] RRR | 0.225 | Success 4.8 yrs  Failure 7.8 yrs | -0.44 (-0.83, -0.04) |
|  | Harigai 2012 [36] | 0.07 | Success 4.4 yrs  Failure 18.1 yrs | -1.44 (-2.63, -0.24) | Brocq 2009 [49] | 0.034 | Success 11.6 yrs  Failure 11.5 yrs | 0.01 (-1.00, 1.02) |
|  | Saleem 2010 [33] | 0.28 | Success median 1.5 yrs  Failure median 6.0 yrs | -0.34 (-0.92, 0.24) | Iwamoto 2014 [51] | 0.087 | Success 7.9 yrs  Failure 7.0 yrs | 0.15 (-0.47, 0.78) |
|  |  |  |  |  | Takai 2020 [60] | 0.04 | Success median 6.9 yrs  Failure median 8.8 yrs | 0.37 (-0.56, 1.31) |
|  |  |  |  |  | Kurasawa 2014 [52] | 0.056 | Success 6.3 yrs  Failure 8.5 yrs | -0.36 (-1.14, 0.42) |
|  |  |  |  |  | Kameda 2021 [61] | 0.075 | Success median 4.1 yrs  Failure median 5.7 yrs | 0.17 (-0.51, 0.85) |
|  |  |  |  |  | Naniwa 2020 [59] | 0.194 | Success median 1.0 yrs  Failure median 1.9 yrs | -0.33 (-0.75, 0.09) |
|  |  |  |  |  | Nagatani 2021 [64] | 0.08 | Success median 5.0 yrs  Failure median 6.5 yrs | -0.57 (-1.23, 0.08) |
|  |  |  |  |  | Ochiai 2021 [62] | 0.095 | Success median 3.0 yrs  Failure median 3.0 yrs | -0.22 (-0.82, 0.38) |
|  |  |  |  |  | Kawashiri 2017 [56] | 0.088 | Success median 3.0 yrs  Failure median 5.0 yrs | -0.50 (-1.13, 0.12) |
| **Summary SMD** |  |  |  | **-0.40 (-0.72, -0.08) p=.02** |  |  |  | **-0.26 (-0.45, -0.07) p=.006** |
| **I^2^** |  |  |  | **4.9** |  |  |  | **0** |
|  |  |  |  |  |  |  |  |  |
| **Duration of RA, categorical** | Emery 2018 [65] PRIZE | 0.505 | Per year. OR estimated from figure | 1.00 (0.58, 1.70) | Ghiti Moghadam 2019 [69] | 0.646 | Duration ≥ 10 yrs vs < 10 yrs  Longer duration less likely to have success.  Invert OR | 0.50 (0.33, 0.74) |
|  | Tanaka 2020 [43] RRRR | 0.495 | Success 31% ≥ 3yrs  Failure 44% ≥ 3yrs | 0.57 (0.33, 0.99) | Curtis, 2021 [71] | 0.353 | Success more likely with RA dur ≤ 5 yrs vs > 5 yrs in MTX group. Invert OR | 0.18 (0.06, 0.57) |
| **Summary OR** |  |  |  | **0.76 (0.44, 1.31)**  **p=0.32** |  |  |  | **0.35 (0.13, 0.91)**  **p=0.03** |
| **I^2^** |  |  |  | **50.5** |  |  |  | **63.0** |
|  |  |  |  |  |  |  |  |  |
| **Duration of RA, categorical HR** | Van den Broek 2011 [66] BeST | 1.0 | Longer duration less likely to succeed. Invert, and convert mo to yrs | 0.78 (0.69, 0.88) | Kavanaugh 2015 [54] | **-** | No association per text | No data |
|  |  |  |  |  | Yoshida 2016 [55] | 1.0 | HR, per 10 yrs.  Invert HR | 1.05 (0.74, 1.47) |
| **Summary HR** |  |  |  | **0.78 (0.69, 0.88) p<.001** |  |  |  | **1.05 (0.74, 1.47) p=0.77** |
| **I^2^** |  |  |  | **-** |  |  |  | **-** |
|  |  |  |  |  |  |  |  |  |
| **Duration of TNFI treatment prior to discontinuation, continuous** | Tanaka 2015 [39] HONOR | 0.70 | Success 14.9 mo  Failures 18.9 mo | -0.58 (-1.13, -0.02) | Chatzidionysiou  2016 [47] | 0.147 | Success median 12mo  Failure median 45.6 mo | -1.14 (-2.47, 0.18) |
|  | Migliore 2010 [34] | 0.295 | Success 7.0 mo  Failure 7.2 mo | -0.13 (-0.99, 0.72) | Brocq 2009 [49] | 0.177 | Success 56 mo  Failure 35 mo | 1.19 (0.11, 2.27) |
|  |  |  |  |  | Takai 2020 [60] | 0.195 | Success median 52 mo  Failure median 40 mo | 0.47 (-0.46, 1.42) |
|  |  |  |  |  | Kurasawa 2014 [52] | 0.216 | Estimated from number of infusions (q8wk)  Success 33.4 mo  Failure 27.8 mo | 0.36 (-0.42, 1.14) |
|  |  |  |  |  | Naniwa 2020 [59] | 0.26 | Success 24.7 mo  Failure 33.1mo | -0.56 (-0.99, -0.13) |
| **Summary SMD** |  |  |  | **-0.45 (-0.91, 0.01) p=.06** |  |  |  | **0.06 (-0.66, 0.79) p=0.86** |
| **I^2^** |  |  |  | **0** |  |  |  | **73.4** |
|  |  |  |  |  |  |  |  |  |
| **Duration of TNFI treatment prior to discontinuation,**  **categorical** | - | - | - | - | Curtis 2021 [71] | 0.667 | Longer use associated with success, per yr  All pts (not only those in Discontinue group). | 1.12 (1.03, 1.25) |
|  |  |  |  |  | Van Vollenhoven 2016 [44] | 0.332 | Longer use more likely to succeed, per yr All pts (not only those in Discontinue group). | 1.47 (1.04, 2.04) |
| **Summary OR** |  |  |  |  |  |  |  | **1.22 (0.95, 1.57) p=0.12** |
| **I^2^** |  |  |  |  |  |  |  | **57.9** |
|  |  |  |  |  |  |  |  |  |
| **Duration of TNFI treatment prior to discontinuation,**  **Categorical HR** | Van den Broek 2011 [66] BeST | 1.0 | Shorter use associated with success. Invert, convert mo to yr | 0.54 (0.41, 0.78) | **-** | **-** | **-** | **-** |
| **Summary HR** |  |  |  | **0.54 (0.41, 0.78)**  **p=.002** |  |  |  |  |
| **I^2^** |  |  |  | **-** |  |  |  |  |
|  |  |  |  |  |  |  |  |  |
|  |  |  |  |  |  |  |  |  |
| **Length of remission at time of discontinuation** | - | - | - | - | Brocq 2009 [49] | 0.109 | Success 35 mo  Failure 14.5 mo | 1.35 (0.25, 2.45) |
|  |  |  |  |  | Iwamoto 2014 [51] | 0.194 | Success 22.3 mo  Failure 24.9 mo | -0.16 (0.79, 0.47) |
|  |  |  |  |  | Takai 2020 [60] | 0.13 | Success median 41.5 mo  Failure median 24.1 mo | 0.63 (-0.31, 1.59) |
|  |  |  |  |  | Kameda 2021 [61] | 0.18 | Success median 24 mo  Failures 18 mo | 0.32 (-0.36, 1.01) |
|  |  |  |  |  | Kawashiri 2017 [56] | 0.195 | Success median 9 mo  Failures median 8 mo | -0.42 (-1.05, 0.20) |
|  |  |  |  |  | Nagatani 2021 [64] | 0.19 | Success median 41.5 mo  Failure median 47.5 mo | -0.21 (-0.86, 0.43) |
| **Summary SMD** |  |  |  |  |  |  |  | **0.13 (-0.31, 0.57) p=0.56** |
| **I^2^** |  |  |  |  |  |  |  | **53.6** |
|  |  |  |  |  |  |  |  |  |
| **Time to achieve remission with TNFi, continuous** | - | - | - | - | Naniwa 2020 [59] | 0.60 | Success median 2.3 mo  Failure median 9.7 mo | -0.33 (-0.75, 0.09) |
|  |  |  |  |  | Takai 2020 [60] | 0.122 | Success median 3.8 mo  Failure median 8.0 mo | -0.48 (-1.43, 0.45) |
|  |  |  |  |  | Kawashiri 2017 [56] | 0.277 | Success median 3.0 mo  Failure median 3.0 mo | -0.37 (-1.00, 0.25) |
| **Summary SMD** |  |  |  |  |  |  |  | **-0.36 (-0.69, -0.03) p=0.03** |
| **I^2^** |  |  |  |  |  |  |  | **0** |
|  |  |  |  |  |  |  |  |  |
| **Time to achieve remission with TNFi, categorical** | Migliore 2010 [34] | 1.0 | Success 80% within 6 mo  Failure 45% within 6 mo | 4.66 (0.70, 31.03) | - | - | - | - |
|  | Migliore 2011 [35] | - | Unable to calculate | No data |  |  |  |  |
| **Summary OR** |  |  |  | **4.66 (0.70, 31.03) p 0.82** |  |  |  |  |
| **I^2^** |  |  |  | **-** |  |  |  | **-** |
|  |  |  |  |  |  |  |  |  |
| **Time to achieve remission with TNFi, categorical HR** | - | - | - | - | Yoshida 2016 [55] | 1.0 | HR inverted. Longer time to remission less likely to succeed. | 0.67 (0.47, 0.95) |
| **Summary HR** |  |  |  |  |  |  |  | **0.67 (0.47, 0.95) p 0.03** |
| **I^2^** |  |  |  |  |  |  |  | **-** |
|  |  |  |  |  |  |  |  |  |
| **Type of TNFi (Monoclonal Antibody versus Etanercept)** | Migliore 2011 [35] | - | Percentages do not add; omit | No data | Ghiti Moghadam 2019 [69] | 0.46 | Only OR provided | 2.26 (1.53, 3.34) |
|  |  |  |  |  | Iwamoto 2014 [51] | 0.026 | Success 100% mAB  Failure 75% mAb | 14.36 (0.67, 307.35) |
|  |  |  |  |  | Naniwa 2020 [59] | 0.208 | Success 75% mAB  Failure 63% mAB | 1.79 (0.71, 4.47) |
|  |  |  |  |  | Nagatani 2021 [64] | 0.122 | Success 42% mAB  Failure 42% mAB | 1.02 (0.27, 3.80) |
|  |  |  |  |  | Kameda 2021 [61] | 0.089 | Success 58% mAB  Failure 64% mAB | 0.77 (0.15, 3.79) |
|  |  |  |  |  | Ochiai 2021 [62] | 0.091 | Success 72% mAB  Failures 82% mAB | 0.57 (0.12, 2.72) |
| **Summary OR** |  |  |  |  |  |  |  | **1.64 (0.98, 2.74) p=.06** |
| **I^2^** |  |  |  |  |  |  |  | **26.4** |
|  |  |  |  |  |  |  |  |  |
| **Weekly MTX dose, continuous** | Tanaka 2015 [39] HONOR | 0.50 | Success 8.1 mg  Failures 8.6 mg | -0.21 (-0.76, 0.32) | Chatzidionysiou  2016 [47] | 0.024 | Success median 20 mg  Failure median 18.75 mg | 0.03 (-1.22, 1.30) |
|  | Tanaka 2016 {41] HOPEFUL-2 | 0.50 | Success 7.1 mg  Failures 7.4 mg | -0.15 (-0.70, 0.39) | Iwamoto 2014 [51] | 0.098 | Success median 8 mg  Failure median 8 mg | -0.24 (-0.88, 0.38) |
|  |  |  |  |  | Kameda 2021 [61] | 0.085 | Success median 8 mg  Failure median 9 mg | 0.04 (-0.63, 0.73) |
|  |  |  |  |  | Kurasawa 2014 [52] | 0.064 | Success 7.6 mg  Failure 7.5 mg | 0.08 (-0.70, 0.86) |
|  |  |  |  |  | Nagatani 2021 [64] | 0.092 | Success median 6 mg  Failure median 5 mg | 0.30 (-0.34, 0.96) |
|  |  |  |  |  | Naniwa 2020 [59] | 0.22 | Success median 12.5 mg  Failure median 12 mg | 0.28 (-0.13, 0.71) |
|  |  |  |  |  | Ochiai 2021 [62] | 0.107 | Success median 8 mg  Failure median 8 mg | -0.28 (-0.89, 0.32) |
|  |  |  |  |  | Takai 2020 [60] | 0.045 | Success median 8 mg  Failure median 8 mg | 0 (-0.93, 0.93) |
|  |  |  |  |  | Tanaka 2010 [50] RRR | 0.26 | Success 7.9 mg  Failure 7.8 mg | 0.04 (-0.34, 0.43) |
| **Summary SMD** |  |  |  | **-0.18 (-0.57, 0.20)**  **p=0.34** |  |  |  | **0.05 (-0.14, 0.25)**  **p= 0.57** |
| **I^2^** |  |  |  | **0** |  |  |  | **0** |
|  |  |  |  |  |  |  |  |  |
| **Weekly MTX dose, categorical** | Tanaka 2020 [43] RRRR |  | Combined arms  OR MTX≥ 10 mg versus < 10 mg | 0.78 (0.46, 1.34) | **`** | **`** | ` | ` |
| **Summary OR** |  |  |  | **0.78 (0.46, 1.34) p=0.37** |  |  |  |  |
| **I^2^** |  |  |  | **-** |  |  |  |  |
|  |  |  |  |  |  |  |  |  |
|  |  |  |  |  |  |  |  |  |
| **Glucocorticoid use, OR** | Harigai 2012 [36] | 0.40 | Success 75% users  Failures 33% users | 6.00 (0.49, 73.4) | Iwamoto 2014 [51] | 0.07 | Success 16.7% users  Failures 12.5% users | 1.40 (0.22, 8.72) |
|  | Tanaka 2016 [41] HOPEFUL-2 | 0.59 | Success 22% users  Failures 37% users | 0.46 (0.14, 1.51) | Kameda 2021 [61] | 0.04 | Success 7.1% users  Failures 10% users | 0.69 (0.05, 8.47) |
|  |  |  |  |  | Kurasawa 2014 [52] | 0.07 | Success 20% users  Failures 23.5% users | 0.81 (0.12, 5.50) |
|  |  |  |  |  | Nagatani 2021 [64] | 0.03 | Success 0% users  Failures 15.3% users | 0.17 (0.009, 1.11) |
|  |  |  |  |  | Naniwa 2020 [59] | 0.24 | Success 41.9% users  Failures 60.6% users | 0.46 (0.19, 1.11) |
|  |  |  |  |  | Ochiai 2021 [62] | 0.14 | Success 68% users  Failures 38.8% users | 3.33 (0.94, 11.85) |
|  |  |  |  |  | Takai 2020 [60] | 0.06 | Success 75% users Failures 70% users | 1.28 (0.15, 10.45) |
| **Summary OR** |  |  |  | **1.30 (0.11, 15.17)**  **p=0.84** |  |  |  | **0.93 (0.46, 1.91) p=0.85** |
| **I^2^** |  |  |  | **69.4** |  |  |  | **23.6** |
|  |  |  |  |  |  |  |  |  |
| **Glucocorticoid use, HR** | **-** | **-** | **-** | **-** | Yoshida 2016 [55] | 1.0 | HR inverted | 0.56 (0.29, 1.08) |
| **Summary HR** |  |  |  |  |  |  |  | **0.56 (0.29, 1.08) p=0.09** |
|  |  |  |  |  |  |  |  |  |
| **RF value,**  **continuous** | Tanaka 2016 [41] HOPEFUL-2 | 0.405 | Success 40.7  Failure 52.4 | -0.27 (-0.76, 0.33) | Tanaka 2010 [50] RRR | 0.727 | Success 225.7  Failure 197.9 | 0.05 (-0.33, 0.44) |
|  | Tanaka 2015 [39] HONOR | 0.402 | Success 58.6  Failure 30.9 | 0.51 (-0.03, 1.06) | Kurasawa 2014 [52] | 0.272 | Success 378.5  Failure 48.6 | 0.57 (-0.21, 1.37) |
|  | Harigai 2012 [36] | 0.192 | Success 78.8  Failure 259.8 | -0.44 (-1.56, 0.66) |  |  |  |  |
| **Summary SMD** |  |  |  | **0.03 (-0.55, 0.61) p=0.91** |  |  |  | **0.19 (-0.26, 0.65) p=.40** |
| **I^2^** |  |  |  | **54.2** |  |  |  | **25.6** |
|  |  |  |  |  |  |  |  |  |
| **RF positive vs negative,**  **categorical** | Emery 2018 [65] PRIZE | 0.092 | OR estimated from figure | 1.00 (0.2, 5.00) | Ghiti Moghadam 2019 [69] | 0.558 | Only OR provided. Invert value for RF negative. | 0.87 (0.56, 1.33) |
|  | Tanaka 2020 [43] RRRR | 0.907 | Success 44% pos  Failure 53% pos  Rf pos less likely to succeed | 0.71 (0.42, 1.18) | Brocq 2009 [49] | 0.017 | Success 80% pos  Failure 47% pos | 4.57 (0.40, 51.13) |
|  |  |  |  |  | Iwamoto 2014 [51] | 0.031 | Success 79% pos  Failures 87% pos | 0.54 (0.09, 3.21) |
|  |  |  |  |  | Takai 2020 [60] | 0.02 | Success 75% pos  Failure 80% pos | 0.75 (0.08, 6.95) |
|  |  |  |  |  | Kameda 2021 [61] | 0.037 | Success 64% pos  Failure 85% pos | 0.31 (0.06, 1.64) |
|  |  |  |  |  | Naniwa 2020 [59] | 0.057 | Success 77% pos  Failure 91% pos | 0.34 (0.09, 1.29) |
|  |  |  |  |  | Nagatani 2021 [64] | 0.018 | Success 93% pos  Failure 88% (rf or acpa pos) | 1.69 (0.16, 18.01) |
|  |  |  |  |  | Kawashiri 2017 [56] | 0.063 | Success 57% pos  Failure 58% pos | 0.97 (0.27, 3.40) |
|  |  |  |  |  | Ochiai 2021 [62] | 0.055 | Success 72% pos  Failure 72% pos | 0.99 (0.25, 3.82) |
|  |  |  |  |  | Curtis, 2021 [71] | 0.14 | Data on whole cohort, not only discontinuation group | 0.46 (0.19, 1.07) |
| **Summary OR** |  |  |  | **0.73 (0.45, 1.20)**  **p=0.21** |  |  |  | **0.75 (0.54, 1.03) p=.08** |
| **I^2^** |  |  |  | **0** |  |  |  | **0** |
|  |  |  |  |  |  |  |  |  |
| **RF positive vs negative, categorical HR** | Van den Broek 2011 [66] BeST | 1.0 | Invert HR | 0.83 (0.41, 1.67) | Kavanaugh 2015 [54] |  | RF positive less likely to have successful discontinuation | No data |
| **Summary HR** |  |  |  | **0.83 (0.41, 1.67)**  **p=0.62** |  |  |  | **-** |
| **I^2^** |  |  |  | **-** |  |  |  |  |
|  |  |  |  |  |  |  |  |  |
|  |  |  |  |  |  |  |  |  |
| **ACPA values, continuous** | Tanaka 2016 [41] HOPEFUL-2 | 1.0 | Success 190.8 Failure 368.9 | -0.27 (-0.82, 0.27) | **-** | **-** | **-** | **-** |
| **Summary SMD** |  |  |  | **-0.27 (-0.82, 0.27)**  **p=0.35** |  |  |  |  |
| **I^2^** |  |  |  | **-** |  |  |  |  |
|  |  |  |  |  |  |  |  |  |
| **ACPA positive versus negative, categorical** | Emery 2018 [65] PRIZE | 1.0 | OR estimated from figure | 0.40 (0.11, 1.40) | Ghiti Moghadam 2019 [69] | 0.62 | Only OR provided. invert | 0.94 (0.62, 1.42) |
|  |  |  |  |  | Brocq 2009 [49] | 0.025 | Success 40% pos  Failure 53% pos | 0.58 (0.07, 4.56) |
|  |  |  |  |  | Iwamoto 2014 [51] | 0.051 | Success 71% pos  Failure 75% pos | 0.81 (0.19, 3.39) |
|  |  |  |  |  | Takai 2020 [60] | 0.017 | Success 87% pos  Failure 70% pos | 3.00 (0.24, 36.32) |
|  |  |  |  |  | Kameda 2021 [61] | 0.045 | Success 57% pos  Failure 80% pos | 0.33 (0.07, 1.52) |
|  |  |  |  |  | Naniwa 2020 [59] | 0.04 | Success 85% pos  Failure 94% pos | 0.38 (0.07, 1.87) |
|  |  |  |  |  | Kawashiri 2017 [56] | 0.017 | Success 90% pos  Failure 94% pos | 0.52 (0.04, 6.33) |
|  |  |  |  |  | Ochiai 2021 [62] | 0.035 | Success 63% pos  Failure 86% pos | 0.26 (0.04, 1.50) |
|  |  |  |  |  | Curtis, 2021 [71] | 0.141 | Data on whole cohort, not only discontinuation group | 1.41 (0.59, 3.36) |
| **Summary OR** |  |  |  | **0.40 (0.11, 1.40) p=0.16** |  |  |  | **0.86 (0.62, 1.20) p=0.39** |
| **I^2^** |  |  |  | **-** |  |  |  | **0** |
|  |  |  |  |  |  |  |  |  |
| **ACPA positive vs negative, categorical HR** | Van den Broek 2011 [66] BeST | 1.0 | Invert HR | 0.66 (0.34, 1.25) | **-** | **-** | **-** | **-** |
| **Summary HR** |  |  |  | **0.66 (0.34, 1.25) p=0.22** |  |  |  |  |
| **I^2^** |  |  |  | **-** |  |  |  |  |
|  |  |  |  |  |  |  |  |  |
|  |  |  |  |  |  |  |  |  |
| **HLA shared epitope** | Van den Broek 2011 [66] BeST | 1.0 | Invert HR | 0.25 (0.09, 0.71) | **-** | **-** | **-** | **-** |
| **Summary HR** |  |  |  | **0.25 (0.09, 0.71) p=0.008** |  |  |  |  |
| **I^2^** |  |  |  | **-** |  |  |  |  |
|  |  |  |  |  |  |  |  |  |
|  |  |  |  |  |  |  |  |  |
| **Radiographic damage,**  **continuous (Sharp scores)** | Tanaka 2016 [41] HOPEFUL-2 | 1.0 | Success 14.2  Failure 25.9 | -0.47 (-1.02, 0.07) | Naniwa 2020 [59] | 0.557 | Success median 5  Failure median 8 | -0.49 (-0.91, -0.06) |
|  |  |  |  |  | Kurasawa 2014 [52] | 0.17 | Success 54.9  Failure 61.0 | -0.07 (-0.85. 0.71) |
|  |  |  |  |  | Tanaka 2010 [50] RRR | 0.27 | Success 46.9  Failure 97.2 | -0.79 (-1.41, -0.17) |
| **Summary SMD** |  |  |  | **-0.47 (-1.02, 0.07)**  **p=0.11** |  |  |  | **-0.50 (-0.82, -0.17) p=0.002** |
| **I^2^** |  |  |  | **-** |  |  |  | **1.3** |
|  |  |  |  |  |  |  |  |  |
| **Radiographic damage,**  **categorical** | Emery 2018 [65] PRIZE | 0.852 | OR estimated from figure, per TSS unit | 0.95 (0.72, 1.25) | Ghiti Moghadam 2019 [69] | 0.16 | Erosive versus nonerosive. Only OR provided. | 0.61 (0.40, 0.92) |
|  | Harigai 2012 [36] | 0.147 | Success 100% Steinbrocker III/IV  Failure 60% Steinbrokcer III/IV | 6.15 (0.28, 134.97) | Nagatani 2021 [64] | 0.01 | Success 14% Steinbrocker III/IV  Failure 30% Steinbrocker III/IV | 0.37 (0.06, 2.08) |
|  |  |  |  |  | Takai 2020 [60] | 0.007 | Success 37% Steinbrocker III/IV  Failure 60% Steinbrocker III/IV | 0.40 (0.05, 2.70) |
|  |  |  |  |  | Van Vollenhoven 2016 [44] | 0.82 | Per 10 unit TSS.  Data from entire cohort, not only discontinuation group | 0.83 (0.70, 0.98) |
| **Summary OR** |  |  |  | **1.25 (0.34, 4.59)**  **p=0.73** |  |  |  | **0.78 (0.66, 0.92)**  **p=0.004** |
| **I^2^** |  |  |  | **28.4** |  |  |  | **2.3** |
|  |  |  |  |  |  |  |  |  |
| **Radiographic damage, categorical HR** | Van den Broek 2011 [66] BeST | 1.0 | Higher score less likely to succeed, per TSS unit, invert | 0.98 (0.96, 1.00) | **-** | **-** | **-** | **-** |
| **Summary HR** |  |  |  | **0.98 (0.96, 1.00)**  **p=0.05** |  |  |  |  |
| **I^2^** |  |  |  | **-** |  |  |  |  |
|  |  |  |  |  |  |  |  |  |
| **BMI,**  **continuous** | **-** | **-** | **-** | **-** | Naniwa 2020 [59] | 1.0 | Success median 21.2  Failure median 19.8 | 0.32 (-0.09, 0.75) |
| **Summary SMD** |  |  |  |  |  |  |  | **0.32 (-0.09, 0.75)**  **p=0.14** |
| **I^2^** |  |  |  |  |  |  |  | **-** |
|  |  |  |  |  |  |  |  |  |
| **BMI,**  **categorical** | - | - | - | - | Ghiti Moghadam 2019 [69] | 0.753 | Over/underweight versus normal BMI. Only OR provided | 0.74 (0.50, 1.08) |
|  |  |  |  |  | Curtis, 2021 [71] | 0.246 | Obese versus not obese BMI. Entire cohort, not only discontinuation group | 0.63 (0.32, 1.22) |
| **Summary OR** |  |  |  |  |  |  |  | **0.71 (0.51, 0.99) p=.04** |
| **I^2^** |  |  |  |  |  |  |  | **0** |
|  |  |  |  |  |  |  |  |  |
| **BMI categorical HR** | Van den Broek 2011 [66] BeST | 1.0 | HR, per unit.  invert | 0.96 (0.89, 1.04) | Kavanaugh 2015 [54] | 1.0 | Overweight/obese versus normal BMI | 0.80 (0.66, 0.96) |
| **Summary HR** |  |  |  | **0.96 (0.89, 1.04) p=0.69** |  |  |  | **0.80 (0.66, 0.96)**  **p=0.02** |
| **I^2^** |  |  |  | **-** |  |  |  |  |
|  |  |  |  |  |  |  |  |  |
|  |  |  |  |  |  |  |  |  |
| **Smoking** | **-** | **-** | **-** | **-** | Naniwa 2020 [59] | 1.0 | Current smoker versus not | 0.63 (0.21, 1.88) |
| **Summary OR** |  |  |  |  |  |  |  | **0.63 (0.21, 1.88) p=0.41** |
| **I^2^** |  |  |  |  |  |  |  | **-** |
|  |  |  |  |  |  |  |  |  |
| **Smoking** | Van den Broek 2011 [66] BeST | 1.0 | Invert HR | 0.41 (0.23, 0.71) | Kavanaugh 2015 [54] | 1.0 | Ever smoker versus never smoker | 0.84 (0.70, 1.00) |
| **Summary HR** |  |  |  | **0.41 (0.23, 0.71) p=.002** |  |  |  | **0.83 (0.70, 0.99) p=0.04** |
| **I^2^** |  |  |  | **-** |  |  |  | **-** |
|  |  |  |  |  |  |  |  |  |
|  |  |  |  |  |  |  |  |  |
| **HAQ, continuous** | Harigai 2012 [36] | 0.089 | Success 0.2  Failure 0.3 | -0.17 (-1.27, 0.93) | Chatzidionysiou  2016 [47] | 0.039 | Success median 0.4  Failure median 0.4 | 0 (-1.26, 1.26) |
|  | Tanaka 2015 [39] HONOR | 0.32 | Success 0.18  Failure 0.26 | -0.25 (-0.80, 0.29) | Brocq 2009 [49] | 0.06 | Success 0.58  Failure 0.4 | 0.29 (-0.71, 1.31) |
|  | Tanaka 2016 [41] HOPEFUL-2 | 0.32 | Success 0.199  Failure 0.219 | -0.06 (-0.61, 0.48) | Iwamoto 2014 [51] | 0.155 | Success median 0.1  Failure median 0.3 | 0.15 (-0.47, 0.79) |
|  | Saleem 2010 [33] | 0.27 | Success median 0  Failure median 0.5 | -0.80 (-1.41, -0.20) | Kameda 2021 [61] | 0.133 | Success median 0  Failure median 0 | 0 (-0.68, 0.68) |
|  |  |  |  |  | Kawashiri 2017 [56] | 0.162 | Success median 0  Failure median 0 | 0 (-0.62, 0.62) |
|  |  |  |  |  | Kurasawa 2014 [52] | 0.10 | Success 0.2  Failure 0.4 | -0.44 (-1.23, 0.34) |
|  |  |  |  |  | Naniwa 2020 [59] | 0.35 | Success median 0  Failure median 0 | 0 (-0.42, 0.42) |
|  |  |  |  |  |  |  |  |  |
| **Summary SMD** |  |  |  | **-0.33 (-0.67, 0)**  **p=.05** |  |  |  | **0 (-0.25, 0.24)**  **p=.99** |
| **I^2^** |  |  |  | **12.1** |  |  |  | **0** |
|  |  |  |  |  |  |  |  |  |
| **HAQ, categorical** | Emery 2018 [65] PRIZE | 1.0 | Estimated from figure, per 1 unit increase | 0.98 (0.60, 1.60) | - | - | - | - |
| **Summary OR** |  |  |  | **0.98 (0.60, 1.60)**  **p=0.94** |  |  |  |  |
| **I^2^** |  |  |  | **-** |  |  |  | **-** |
|  |  |  |  |  |  |  |  |  |
| **HAQ categorical HR** | Van den Broek 2011 [66] BeST | 1.0 | HR, per 1 unit increase  invert | 0.66, (0.34, 1.25) | Kavanaugh 2015 [54] | 1.0 | HR, per 1 unit increase, invert | 0.82 (0.69, 0.97) |
| **Summary HR** |  |  |  | **0.66, (0.34, 1.25) p=0.22** |  |  |  | **0.82 (0.69, 0.97)**  **p=0.03** |
| **I^2^** |  |  |  | **-** |  |  |  | **-** |
|  |  |  |  |  |  |  |  |  |
|  |  |  |  |  |  |  |  |  |
| **Disease activity,**  **continuous** | Harigai 2012 [36] | 0.166 | Success DAS28 1.5  Failure DAS28 1.7 | -0.52 (-1.63, 0.59) | Chatzidionysiou  2016 [47] | 0.063 | Success DAS28 median 1.7  Failure DAS28 median 1.7 | -0.74 (-2.03, 0.55) |
|  | Migliore 2010 [34] | 0.217 | Success DAS28 mean 1.75  Failure DAS28 mean 1.69 | 0.16 (-0.71, 1.05) | Iwamoto 2014 [51] | 0.147 | Success DAS28 median 2.0  Failure DAS28 median 1.9 | -0.18 (-0.81, 0.44_ |
|  | Tanaka 2015 [39] HONOR | 0.30 | Success DAS28 1.7  Failure DAS28 2.2 | -1.09 (-1.67, -0.50) | Kameda 2021 [61] | 0.135 | Success DAS28 median 1.6  Failure DAS28 median 2.3 | -0.61 (-1.31, 0.08) |
|  | Tanaka 2016 [41] HOPEFUL-2 | 0.31 | Success DAS28 2.2  Failure DAS28 3.0 | -1.13 (-1.70, -0.55) | Kawashiri 2017 [56] | 0.149 | Success DAS28 median 1.57  Failure DAS28 median 1.73 | -0.05 (-0.67, 0.56) |
|  | Yamanaka 2016 [27] | - | Lower SDAI associated with successful discontinuation | No data | Kurasawa 2014 [52] | 0.117 | Success DAS28 1.4  Failure DAS28 1.8 | -0.75 (-1.56, 0.05) |
|  |  |  |  |  | Nagatani 2021 [64] | 0.138 | Success DAS28 median 1.29  Failure DAS28 median 1.09 | 0.87 (0.19, 1.55) |
|  |  |  |  |  | Ochiai 2021 [62] | 0.152 | Success DAS28 median 1.93  Failure DAS28 median 2.15 | -0.17 (-0.78, 0.43) |
|  |  |  |  |  | Takai 2020 [60] | 0.096 | Success DAS28 median 1.56  Failure DAS28 median 1.88 | -0.61 (-1.56, 0.33) |
|  |  |  |  |  | Curtis 2021 [46] | 0 | Lower SDAI associated with successful discontinuation. Data on entire cohort, not only those who discontinued | No data |
| **Summary SMD** |  |  |  | **-0.73 (-1.30, -0.16) p=0.02** |  |  |  | **-0.21 (-0.59, 0.16) p=0.26** |
| **I^2^** |  |  |  | **57.4** |  |  |  | **51.7** |
|  |  |  |  |  |  |  |  |  |
| **Disease activity, categorical** | Emery 2018 [65] PRIZE | 0.527 | Estimated from figure, per 1 unit increase in DAS28 | 0.95 (0.60, 1.50) | Tanaka 2010 [50] RRR | 0.325 | DAS28 ≥ 2.225 versus < 2.225 | 0.15 (0.06, 0.37) |
|  | Migliore 2011 [35] | - | Unable to calculate | No data | Ghiti Moghadam 2019 [69] | 0.391 | DAS28 > 1.98 versus ≤ 1.98 | 0.82 (0.56, 1.20) |
|  | Tanaka 2020 [43] RRRR | 0.472 | SDAI ≥ 26 associated with lower likelihood of success | 0.49 (0.28, 0.85) | Naniwa 2020 [59] | 0.283 | DAS28 > 2.6 versus ≤ 2.6 | 0.76 (0.24, 2.37) |
| **Summary OR** |  |  |  | **0.70 (0.37,1.32)**  **p=0.27** |  |  |  | **0.47 (0.16, 1.38)**  **p=0.17** |
| **I^2^** |  |  |  | **69.0** |  |  |  | **83.0** |
|  |  |  |  |  |  |  |  |  |
| **Disease activity, categorical HR** | Smolen 2018 [67] PRESERVE | 0.533 | HR per 1 unit change in DAS28.  Risk of loss of remission higher with higher DAS28 at wk 36 | 0.57 (0.39, 0.83) | Kavanaugh 2015 [54] | 0.821 | HR per 1 unit change in CDAI. Higher CDAI associated with lower likelihood of success. | 0.78 (0.66, 0.92) |
|  | Van den Broek 2011 [66] BeST | 0.466 | HR per 1 unit change in DAS28.  invert | 0.91 (0.58, 1.42) | Yoshida 2016 [55] | 0.178 | HR per 1 unit change in CDAI. Higher CDAI associated with lower likelihood to succeed. | 0.66 (0.46, 0.94) |
| **Summary HR** |  |  |  | **0.71 (0.45, 1.12)**  **p=0.14** |  |  |  | **0.76 (0.65, 0.88) p< 0.01** |
| **I^2^** |  |  |  | **59.6** |  |  |  | **0** |
|  |  |  |  |  |  |  |  |  |
|  |  |  |  |  |  |  |  |  |
| **MBDA,**  **categorical** | Hirata 2016 [68] HONOR | 1.0 | MBDA > 44 0/5 (0%) success  MBDA ≤ 44 19/37 (51%) success | 0.08 (0.004, 1.67) | Ghiti Moghadam 2019 [69] | - | MBDA > 44 versus ≤ 44. Only OR provided. Invert. | 0.43 (0.24, 0.75) |
| **Summary OR** |  |  |  | **0.08 (0.004, 1.67) p=0.20** |  |  |  | **0.43 (0.24, 0.75) p=0.003** |
| **I^2^** |  |  |  | **-** |  |  |  | **-** |
|  |  |  |  |  |  |  |  |  |
|  |  |  |  |  |  |  |  |  |
| **Ultrasound Grey scale,**  **continuous** | Saleem 2010 [33] | 1.0 | Success median 4  Failure median 5 | -0.45 (-1.34, 0.43) | Iwamoto 2014 [51] | 0.295 | Success median 3  Failure median 13 | -0.13 (-0.77, 0.49) |
|  |  |  |  |  | Okano 2019 [72] | - | Baseline US did not predict relapse | No data |
|  |  |  |  |  | Kameda 2021 [61] | 0.253 | Success median 8  Failure median 6 | 0.09 (-0.58, 0.78) |
|  |  |  |  |  | Kawashiri 2017 [56] | 0.307 | Success median 2  Failure median 2 | 0 (-0.62, 0.62) |
|  |  |  |  |  | Amano 2017 [73] | 0.143 | Success 2.0  Failure 2.0 | 0 (-0.91, 0.91) |
| **Summary SMD** |  |  |  | **-0.45 (-1.34, 0.43) p=0.25** |  |  |  | **-0.02 (-0.36, 0.33) p=0.93** |
| **I^2^** |  |  |  | **-** |  |  |  | **0** |
|  |  |  |  |  |  |  |  |  |
| **Ultrasound Power Doppler, continuous** | Saleem 2010 [33] | 1.0 | Success median 0  Failure median 0 | -0.08 (-0.96, 0.80) | Iwamoto 2014 [51] | 0.279 | Success median 0  Failure median 2 | -0.79 (-1.44, -0.13) |
|  |  |  |  |  | Okano 2019 [72] | - | Baseline US did not predict relapse | No data |
|  |  |  |  |  | Kameda 2021 [61] | 0.26 | Success median 0  Failure median 0 | 0 (-0.68, 0.68) |
|  |  |  |  |  | Kawashiri 2017 [56] | 0.304 | Success median 0  Failure median 0 | -0.29 (-0.91, 0.33) |
|  |  |  |  |  | Amano 2017 [73] | 0.156 | Success 1.5  Failure 1.4 | 0.05 (-0.85, 0.96) |
| **Summary SMD** |  |  |  | **-0.08 (-0.96, 0.80) p=0.97** |  |  |  | **-0.30 (-0.68, 0.07) p=0.12** |
| **I^2^** |  |  |  |  |  |  |  | **13.4** |
|  |  |  |  |  |  |  |  |  |
| **Ultrasound Power Doppler, categorical** | **-** | - | - | - | Lo Monaco 2018 [74] | 0.37 | HR invert.  Doppler ≥ 1 versus 0 | 0.14 (0.03, 0.60) |
|  |  |  |  |  | Lamers-Karnebeek 2017 [75] | 0.63 | HR invert.  Grey Scale>1 or Doppler>0  Success 54%  Failure 69% | 0.58 (0.40, 0.91) |
| **Summary HR** |  |  |  |  |  |  |  | **0.34 (0.09, 1.31) p=0.12** |
| **I^2^** |  |  |  |  |  |  |  | **70.1** |

Supplemental table 10. Predictors of sustained remission in studies of discontinuation of tumor necrosis factor inhibitor

(TNFi) treatment among studies of low or moderate risk of bias.

|  | Induction-Withdrawal studies | | | |  | Maintenance Discontinuation studies | | | |
| --- | --- | --- | --- | --- | --- | --- | --- | --- | --- |
| Predictor | Number of studies | Effect size (SMD, OR or HR) | P | I^2^ |  | Number of studies | Effect size (SMD, OR or HR) | P | I^2^ |
| Older age, continuous, SMD | 1 | -0.48 (-1.03, 0.06) | 0.09 | - |  | 7 | -0.16 (-0.40, 0.07) | 0.17 | 11.3 |
| Older age, categorical, OR | 1 | 0.50 (0.26, 0.95) | 0.04 | - |  | 0 | - | - | - |
| Older age, categorical, HR | 1 | 1.00 (0.98, 1.02) | 0.99 | - |  | 0 | - | - | - |
|  |  |  |  |  |  |  |  |  |  |
| Women vs Men, OR | 1 | OR 0.70 (0.32, 1.50) | 0.38 | - |  | 7 | 0.64 (0.36, 1.12) | 0.12 | 0 |
| Women vs Men, HR | 1 | 0.91 (0.50, 1.66) | 0.97 | - |  | 0 | - | - | - |
|  |  |  |  |  |  |  |  |  |  |
| Duration of RA, continuous, SMD | 2 | -0.33 (-0.73, 0.07) | 0.11 | 0 |  | 7 | -0.27 (-0.48, -0.05) | 0.02 | 0 |
| Duration of RA, categorical, OR | 1 | 1.00 (0.58, 1.70) | 0.93 | - |  | 1 | 0.18 (0.06, 0.57) | 0.003 | - |
| Duration of RA, categorical, HR | 1 | 0.78 (0.69, 0.88) | <0.001 | - |  | 0 | - | - | - |
|  |  |  |  |  |  |  |  |  |  |
| Duration of TNFi treatment prior to discontinuation, continuous, SMD | 1 | -0.58 (-1.13, -0.02) | 0.03 | 0 |  | 3 | 0.24 (-0.77, 1.25) | 0.65 | 82.1 |
| Duration of TNFi treatment prior to discontinuation, categorical, OR | 0 | - | - | - |  | 2 | 1.22 (0.95, 1.57) | 0.12 | 57.9 |
| Duration of TNFi treatment prior to discontinuation, categorical, HR | 1 | 0.54 (0.41, 0.78) | 0.002 | - |  | 0 | - | - | - |
|  |  |  |  |  |  |  |  |  |  |
| Length of remission at time of TNFi discontinuation, continuous, SMD | 0 | - | - | - |  | 4 | 0.20 (-0.36, 0.77) | 0.48 | 57.1 |
|  |  |  |  |  |  |  |  |  |  |
| Time to reach remission with TNFi, continuous, SMD | 0 | - | - | - |  | 1 | -0.33 (-0.75, 0.09) | 0.26 | - |
| Time to reach remission with TNFi, categorical, OR | 0 | - | - | - |  | 0 | - | - | - |
| Time to reach remission with TNFi, categorical, HR | 0 | - | - | - |  | 0 | - | - | - |
|  |  |  |  |  |  |  |  |  |  |
| Type of TNFi: Monoclonal antibody vs Etanercept, OR | 0 | - | - | - |  | 4 | 1.47 (0.72, 2.98) | 0.28 | 6.7 |
|  |  |  |  |  |  |  |  |  |  |
| Methotrexate dose, continuous, SMD | 1 | -0.21 (-0.76, 0.32) | 0.77 | - |  | 6 | 0.10 (-0.11, 0.33) | 0.34 | 0 |
| Methotrexate dose, categorical, OR | 0 | - | - | - |  | 0 | - | - | - |
|  |  |  |  |  |  |  |  |  |  |
| Glucocorticoid use, categorical, OR | 0 | - | - | - |  | 5 | 0.57 (0.29, 1.13) | 0.11 | 0 |
| Glucocorticoid use, categorical, HR | 0 | - | - | - |  | 0 | - | - | - |
|  |  |  |  |  |  |  |  |  |  |
| RF value, continuous, SMD | 1 | 0.51 (-0.03, 1.06) | 0.21 | - |  | 2 | 0.19 (-0.26, 0.65) | 0.40 | 25.6 |
| RF positive vs negative, OR | 1 | 1.00 (0.20, 5.00) | 0.98 | - |  | 5 | 0.62 (0.25, 1.47) | 0.28 | 15.3 |
| RF positive vs negative, HR | 1 | 0.83 (0.41, 1.67) | 0.62 | - |  | 0 | - | - | - |
|  |  |  |  |  |  |  |  |  |  |
| ACPA titer, continuous, SMD | 0 | - | - | - |  | 0 | - | - | - |
| ACPA positive vs negative, OR | 1 | 0.40 (0.11, 1.40) | 0.16 | - |  | 4 | 0.50 (0.22, 1.11) | 0.09 | 0 |
| ACPA positive vs negative, HR | 1 | 0.66 (0.34, 1.25) | 0.22 | - |  | 0 | - | - | - |
|  |  |  |  |  |  |  |  |  |  |
| HLA shared epitope present vs absent, HR | 1 | 0.25 (0.09, 0.71) | 0.008 | - |  | 0 | - | - | - |
|  |  |  |  |  |  |  |  |  |  |
| Radiographic damage, Sharp Score continuous, SMD | 0 | - | - | - |  | 3 | -0.50 (-0.82, -0.17) | 0.002 | 1.3 |
| Radiographic damage, categorical, OR | 1 | 0.95 (0.72, 1.25) | 0.73 | - |  | 2 | 0.83 (0.69, 0.98) | 0.03 | 0 |
| Radiographic damage, categorical, HR | 1 | 0.98 (0.96, 1.00) | 0.05 | - |  | 0 | - | - | - |
|  |  |  |  |  |  |  |  |  |  |
| BMI continuous, SMD | 0 | - | - | - |  | 1 | 0.32 (-0.09, 0.75) | 0.14 | - |
| BMI categorical, OR | 0 | - | - | - |  | 1 | 0.63 (0.32, 1.22) | 0.18 | - |
| BMI categorical, HR | 1 | 0.96 (0.89, 1.04) | 0.69 | - |  | 0 | - | - | - |
|  |  |  |  |  |  |  |  |  |  |
| Smoker vs non-smoker, OR | 0 | - | - | - |  | 1 | 0.63 (0.21, 1.88) | 0.41 | - |
| Smoker vs non-smoker, HR | 1 | 0.41 (0.23, 0.71) | 0.002 | - |  | 0 | - | - | - |
|  |  |  |  |  |  |  |  |  |  |
| HAQ continuous, SMD | 2 | -0.51 (-1.05, 0.03) | 0.07 | 43.2 |  | 5 | 0 (-0.29, 0.28) | 0.99 | 0 |
| HAQ categorical, OR | 1 | 0.98 (0.60, 1.60) | 0.94 | - |  | 0 | - | - | - |
| HAQ categorical, HR | 1 | 0.66 (0.34, 1.25) | 0.22 | - |  | 0 | - | - | - |
|  |  |  |  |  |  |  |  |  |  |
| Disease activity, continuous, SMD | 1 | -1.09 (-1.67, -0.50) | 0.001 | - |  | 4 | -0.15 (-0.88, 0.57) | 0.68 | 76.3 |
| Disease activity, categorical, OR | 1 | 0.95 (0.60, 1.50) | 0.84 | - |  | 2 | 0.33 (0.07, 1.56) | 0.17 | 78.8 |
| Disease activity, categorical, HR | 2 | 0.71 (0.45, 1.12) | 0.14 | 59.6 |  | 0 | - | - | - |
|  |  |  |  |  |  |  |  |  |  |
| MBDA > 44 vs ≤ 44, categorical, OR | 1 | 0.08 (0.004, 1.67) | 0.20 | - |  | 0 | - | - | - |
|  |  |  |  |  |  |  |  |  |  |
| Ultrasound Grey scale, continuous, SMD | 1 | -0.45 (-1.34, 0.43) | 0.25 | - |  | 2 | -0.03 (-0.50, 0.44) | 0.91 | 0 |
|  |  |  |  |  |  |  |  |  |  |
| Ultrasound power Doppler continuous, SMD | 1 | -0.08 (-0.96, 0.80) | 0.97 | - |  | 2 | -0.40 (-1.18, 0.38) | 0.31 | 62.6 |
| Ultrasound power Doppler categorical, HR | 0 | - | - | - |  | 0 | - | - | - |

PRISMA checklist.
